# Toward once-monthly insulin therapy *via* synergy in two pharmacokinetic protractors: Fc-conjugation and fatty acid acylation†

**DOI:** 10.1039/d4cb00078a

**Published:** 2024-06-18

**Authors:** Alexander N. Zaykov, Vasily M. Gelfanov, Tina M. Tagmose, Damien Demozay, Valentina Manfè, Rebecca Rohlfs, Marita Rivir, Diego Perez-Tilve, Brian Finan, Richard D. DiMarchi

**Affiliations:** a Novo Nordisk Research Center Indianapolis Indianapolis IN 46241 USA alexander.n.zaykov@gmail.com; b Novo Nordisk, Global Research Technologies DK-2760 Maaloev Denmark; c Department of Pharmacology and Systems Physiology, University of Cincinnati-College of Medicine Cincinnati OH 45267 USA; d Department of Chemistry, Indiana University Bloomington IN 47405 USA

## Abstract

Pharmacokinetic properties and duration of therapeutic action of a pharmaceutical agent can be significantly extended through the combination of two distinct strategies aimed at increasing plasma half-life: fatty acid acylation and Fc-conjugation. Using insulin as a case study, we demonstrate that a doubly protracted insulin analog produces a substantial prolongation of pharmacodynamic effect to lower blood glucose in STZ-treated mice when compared to the Fc-only counterparts. This enhancement is further corroborated by direct pharmacokinetic measurements in rat and dog models, demonstrating the potential for once-monthly insulin therapy. The results suggest that this approach might have broad application across a diverse spectrum of peptide- and protein-based therapeutics.

## Abstract 2

Pharmacokinetic properties of a peptide hormone are extended *via* the combination of fatty acid acylation and Fc-conjugation, as shown with double protracted insulin analogs. This approach may be expanded to other peptide- and protein-based therapies.

## Graphical abstract

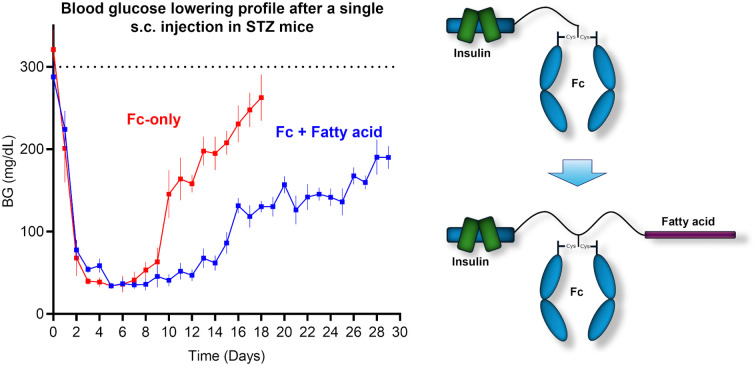

## Introduction

Fc-fusion, PEGylation, and fatty acid acylation are prominent technologies designed to enhance the *in vivo* duration of protein and peptide therapeutics.^1–3^ While other strategies such as carbohydrate conjugation, albumin or transferrin fusions, and protraction using XTEN™, ELP or similar biopolymers have been developed, they are less thoroughly clinically assessed in investigational drugs.^4^ All of these methodologies share a common objective to optimize the pharmacokinetic attributes of the therapeutic agent by reducing its clearance which is often combined with increasing its proteolytic stability. These enhancements lead to a reduced dose requirement to achieve the desired pharmacological effect and decrease the frequency in administration. This latter point is particularly significant for biologics, which typically necessitate injection, posing challenges to patient compliance and potentially impacting the overall success of treatment.^5^

The strategy of peptide lipidation (fatty acid acylation) was initially developed within the context of diabetes research, leading to the development of long-acting insulin analogs like detemir and degludec and subsequently employed with GLP-1 agonists such as liraglutide and semaglutide.^6–10^ More recently, this approach has expanded to create a variety of new therapeutic agents, both approved and in development, including icodec, somapacitan, cagrilintide, tirzepatide, retatrutide, and zilucoplan, among others.^10^ There has been steady progress in the comprehension of how fatty acids interact with albumin, the mechanisms governing the absorption and distribution of lipidated therapeutics, and insights into structure–activity relationships with an emphasis on the protracting entity itself.^9–17^ Optimized lipidated molecules have been engineered to achieve half-lives ranging from 100–300 hours in humans, allowing for once-weekly administration.^10^

The Fc-fusion approach is a well-established strategy that has led to the development of several marketed drugs. Among these, there are highly successful drugs such as etanercept (Enbrel®) for treating various forms of arthritis, aflibercept (Eylea®) for age-related macular degeneration, and dulaglutide (Trulicity®) for type 2 diabetes.^18^ The Fc component modulates the pharmacokinetic properties of the fusion proteins by leveraging the natural properties of IgG antibodies. This occurs through two primary mechanisms. Firstly, the fusion with Fc significantly increases the hydrodynamic size of the molecule, with the Fc protein contributing roughly 50 kDa to the total molecular weight.^4^ Secondly, the presence of Fc leads to a reduction in clearance of the proteins due to active recycling from the endosome, which is facilitated by the neonatal Fc receptor (FcRn).^19,20^ This latter mechanism is key for the prolonged circulating half-life seen in antibodies, and further optimization of Fc domains from native sequences can significantly enhance the circulating half-life that Fc imparts.^21–24^

The recycling process mediated by the FcRn receptor is also a major factor contributing to the extended circulating half-life of albumin, and the direct interactions between FcRn and albumin have been well-documented.^25–30^ It follows that therapeutics which bind to albumin may inherently benefit from this property resulting in an extended systemic exposure.^31^ Moreover, the interaction of FcRn with Fc or albumin is not mutually exclusive, and a ternary complex involving all three components has been characterized (Fig. S1, ESI†).^26^ This raises an intriguing possibility that the simultaneous interaction of Fc and albumin can be integrated to further optimize the FcRn-mediated recycling of therapeutic agents to further extend the duration of action.

Basal insulin therapy aims to regulate blood sugar levels intermediate to prandial periods, notably overnight.^32,33^ An ideal therapy mimics continuous basal insulin production, offering a consistent, peakless profile. To achieve that, basal insulins are typically designed to have slow absorption, as in the cases of insulin NPH, glargine, and degludec, or diminished elimination kinetics, as achieved through protraction methods outlined above and as observed with detemir, degludec, icodec, and insulin efsitora alfa.

Adherence to therapy is a serious challenge in everyday diabetes management using basal insulins.^5^ The need for frequent injections has been linked to lower compliance, leading to inadequate glucose control and diminished treatment outcomes. The issue has encouraged the development of new insulin therapies with prolonged durations of action and reduced injection frequencies,^34,35^ which most recently culminated in the development of two once-weekly insulin analogs: insulin icodec, utilizing a lipidation approach, and insulin efsitora alfa, employing an Fc-fusion approach to improve pharmacokinetic properties.^16,36–38^ The results we report here indicate the approaches are not mutually exclusive and that much longer-acting insulin analogs are feasible by combining the two PK extension strategies.

## Results

### Combination of lipidation and Fc-conjugation prolongs insulin's glucose lowering activity in STZ mice

The synergistic effect of combining two protraction strategies on the pharmacokinetic and pharmacodynamic (PK/PD) profile of an insulin molecule is exemplified in the two analogs, Fc-Ins1 and Fc/FA-Ins1, detailed in Table 1. Both share the same insulin backbone with mutations A14-Glu in the A-chain and B25-His in the B-chain, the changes that reduce receptor-mediated clearance through decreased receptor affinity.^16,39^ The insulin peptide is protracted *via* lysine side-chain acylation at position B29 by a construct containing 10× repeat of the Ado spacer (8-amino-3,6-dioxaoctanoic acid), followed by a lysine residue used for Fc conjugation, and then additional 10× Ado spacer terminating in the fatty acid protractor (-γGlu-C20diacid, see exact structure in ESI†). The Fc component has several mutations that biophysically stabilize the protein to improve recombinant yields: 227Ala, 234Ala, 235Lys, 297Glu, 315Gln, 384Gln, des447 hIgG4 Fc(227–447).^40^ Insulin receptor *in vitro* phosphorylation analysis indicate that both analogs possess diminished potency compared to human insulin, with Fc-Ins1 at approximately 7% and Fc/FA-Ins1 near 3%. This reduced potency is consistent with previous observations involving Fc-conjugated insulins and it is a consequence of A14 and B25 mutations.^40^

**Table 1:** List of insulin compounds and their potency at insulin receptor (IR)

Compound	Insulin^a^	Conjugation position – protractor^b^	EC50 (nM)		Rel. potency^c^ (%)	
			0% HSA	1% HSA	0% HSA	1% HSA
Fc/FA-Ins1	A14E, B25H, desB30	B29 – linker-Fc-linker-lipid	73	122	2.8%	2.0%
			*n* = 7^f^	*n* = 6		
Fc-Ins1	A14E, B25H, desB30	B29 – linker-Fc	24	21	7.3%	7.9%
			*n* = 3	*n* = 3		
FA-Ins1	A14E, B25H, desB30	B29 – linker1-lipid	2.6	67	28%	1.9%
Fc-Ins2	A14E, A21G, B25H, B29R desB30	C-term – linker2-Fc	2.4	4.7	30%	27%
Fc-Ins3	A14E, A21G, B16H, B25H, B29R desB30	C-term – linker2-Fc	4.9	12	17%	11%
Fc-Ins4	A14E, A21G, B16E, B25H, B29R desB30	C-term – linker2-Fc	18	46	4.1%	2.8%
LY3209590	Lilly's Fc-insulin^d^		50	83	1.4%	1.5%
Fc/FA-Ins1(B1)	A14E, B25H, desB30	B1 – linker-Fc-linker-lipid	60	94	3.3%	1.9%
			*n* = 2	*n* = 2		
Fc/FA-Ins5	A14E, A22K, B25H, B29R desB30	A22 – linker-Fc-linker-lipid	12	19	4.9%	7.3%
Fc/FA-Ins6	(A10C-B3C), A14E, B25H, B16H, desB30	B29 – linker-Fc-linker-lipid	770	664	0.24%	0.26%
			*n* = 2	*n* = 2		
Fc/FA-Ins7	A14E, B25H, B16H, desB30	B29 – linker-Fc-linker-lipid	380	336	0.48%	0.51%
			*n* = 2	*n* = 2		
Fc/FA-Ins8	desB30	B29 – linker-Fc-linker-lipid	23	n.d^g^	2.5%	n.d
(Fc/FA)inv-Ins1	A14E, B25H, desB30	B29 – linker-lipid-linker-Fc	34	101	4.1%	1.9%
(Fc/FA)v1-Ins1	A14E, B25H, desB30	B29 – linker3-Fc-linker-lipid	29	28	6.1%	7.0%
			*n* = 2			
(Fc/FA)v2-Ins1	A14E, B25H, desB30	B29 – linker-Fc-linker3-lipid	170	99	1.2%	1.6%.
(Fc/FA)v3-Ins1	A14E, B25H, desB30	B29 – linker-Fc-linker1-lipid	59	n.d.	1.0%	n.d.
(Fc/FA)half-Ins1	A14E, B25H, desB30	B29 – linker-Fc(1/2)-linker-lipid	37	45	3.1%	4.4%
Fc/FA-Ins9	DesDi (desB28, desB30)^e^ – single chain	B29 – linker-Fc-linker-lipid	Inactive	—	—	—
Fc-Ins9	DesDi (desB28, desB30) – single chain	B29 – linker-Fc	Inactive	—	—	—

a A list of insulin backbones used in this study. The corresponding mutations relative to native human insulin are outlined. Mutations provide proteolytic stability needed for extended activity and decrease activity of insulin to reduce active clearance through the receptor.

b Linker represent Ado_10_ (8-amino-3,6-dioxaoctanoic acid), linker1 is Ado_2_, linker2 is (GEQP)_12_-KP, linker3 is Ado_4_, lipid is γGlu-C20diacid motif and “Fc” is an Fc dimer bridged at cysteines *via N*,*N*-bisiodoacetamide-lysine linker. Fc(1/2) represent a structure where only a single Fc unit is conjugated instead of a dimer. See ESI for exact structures.

c EC50 expressed as percent potency relative to human insulin standard used in the same assay. Activity was tested in insulin receptor Y1158 phosphorylation assay in HEK293 cells overexpressing B-isoform of insulin receptor.

d Insulin mutations: A10T, A14D, A21G, A22G, B16E, B25H, desB27-B30, single chain insulin with C-chain: GGGGGGSGGGG.

e Single chain DesDi insulin is an inactive analog of insulin with A and B chain linked between residues B28 and A1.

f *n* = number of repeats used to calculate the reported mean.

g n.d. – not determined.

**Fig. 1:**
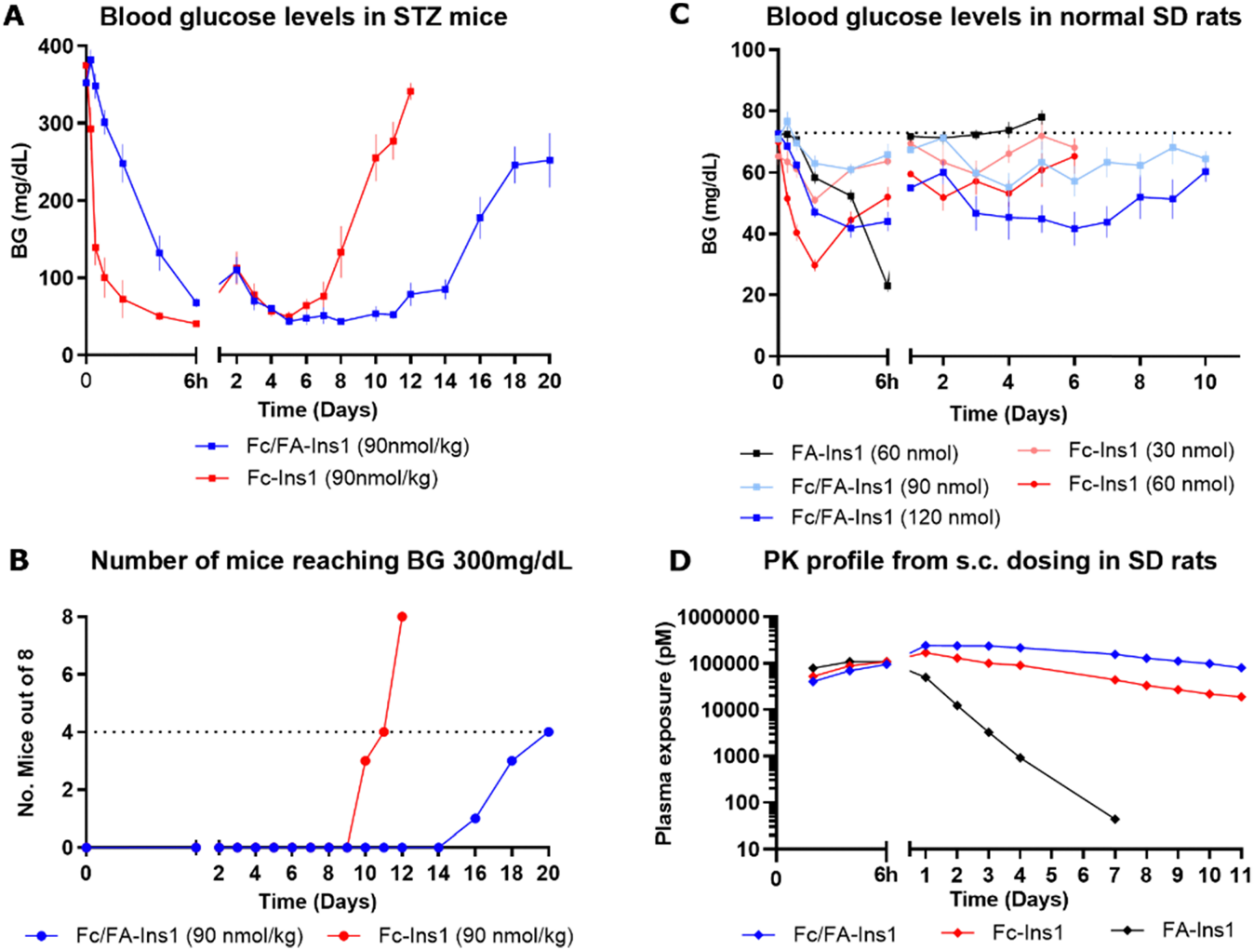
PK and PD profiles of Fc-Ins1 and Fc/FA-Ins1 (protracted by Fc only and doubly protracted with Fc and lipid (γGlu-C20diacid) respectively). (A) Blood glucose lowering in STZ-treated mice following a single 90 nmol per kg s.c. dose of Fc-Ins1 and Fc/FA-Ins1. Once mice reach blood glucose levels above 300 mg dL^−1^, they are removed from the study and no longer registered in the graph. (B) Graph demonstrating the number of mice reaching BG > 300 mg dL^−1^. (C) Plasma glucose levels after s.c. dosing in Sprague Dawley rats. (D) PK profile of Fc/FA-Ins1, Fc-Ins1 and FA-Ins1 analogs. PK parameters are displayed in Table 2. Data are expressed as mean ± SEM.

The analogs were assessed for their glucose-lowering efficacy in STZ-treated mice following a single subcutaneous injection at a dose of 90 nmol kg^−1^ (Fig. 1A). Glucose levels decreased in the initial 6 hours with a noticeable difference between the two insulin analogs. The additional fatty acid moiety in Fc/FA-Ins1 slowed the rate of glucose reduction, suggesting slower absorption or distribution compared to the Fc only protracted analog. Blood glucose levels decreased and stabilized around 50 mg dL^−1^ by day 4. The distinction between the two insulin analogs is most striking in their duration of action as the pharmacological effect of Fc-Ins1 diminishes around day 11, whereas Fc/FA-Ins1 continues to lower glucose levels up to day 20 (Fig. 1B, which illustrates when blood glucose levels in mice return to hyperglycemic values of 300 mg dL^−1^). A similar prolongation of the glucose-lowering profile was observed in normal rats (Fig. 1C). Animals administered Fc/FA-Ins1 exhibited reduced blood glucose for up to 10 days, while glucose levels in rats given Fc-Ins1 returned to normal levels after 5–6 days, aligning with previous observations of an approximate doubling in the duration of action.^40^ By contrast, the fatty acid-protracted insulin analog FA-Ins1 without Fc-conjugation was effective for less than 24 hours (compound #10 in the ref. 41). It is important to note that the duration of action of fatty acid protracted molecules can be misleading when compared to Fc-fusion analogs in rodents due to the much faster turn-over rate of albumin in rodents. The large difference becomes less pronounced when compared in larger animals or humans.

Direct measurement of the pharmacokinetic properties of the insulin analogs in rats revealed approximately a two-fold increase in mean half-life for the doubly protracted peptide (Fig. 1D), aligning with the observed pharmacodynamic effect (Fig. 1C and Table 2). Fc/FA-Ins1 exhibited a half-life of 124 hours compared to 76 hours for the Fc-only analog, and 13 hours for the fatty acid-protracted analog FA-Ins1.

**Table 2:** Pharmacokinetic parameters after subcutaneous dosing in normal Sprague-Dawley rats

Compound	Stat	Dose (nmol kg^−1^)	# of animals	HL_Lambda_z (h)	Cmax (pmol L^−1^)	AUCINF_pred (h*pmol L^−1^)	AUC_%Extrap_pred (%)	Vz_pred (L kg^−1^)	Cl_pred (L h^−1^ kg^−1^)
Fc/FA-Ins1	Mean	30	5	124	2.52 × 10^+05^	6.09 × 10^+07^	26	0.087	4.97 × 10^−04^
	SD			29	1.48 × 10^+04^	6.14 × 10^+06^	7	0.013	5.26 × 10^−05^
Fc-Ins1	Mean	30	5	76	1.69 × 10^+05^	2.12 × 10^+07^	9	0.155	1.42 × 10^−03^
	SD			6	1.68 × 10^+04^	1.28 × 10^+06^	2	0.009	9.10 × 10^−05^
FA-Ins1	Mean	30	5	13	1.11 × 10^+05^	2.92 × 10^+06^	0.3	0.188	1.03 × 10^−02^
	SD			1	1.26 × 10^+04^	1.51 × 10^+05^	0.2	0.021	5.58 × 10^−04^

### Comparison with other Fc-protracted insulin analogs

We aimed to benchmark the Fc/FA-Ins1 analog against other Fc-protracted insulin analogs that have been described previously, amongst them the once-weekly analog LY3209590 (BIF, efsitora alfa). The Fc-Ins2 analog shares the A14-Glu and B25-His mutations present in Ins1 but is further extended from the C-terminus of the A-chain with a (GEQP)12-KP spacer that is conjugated to an Fc protein. The analogs Fc-Ins3 and Fc-Ins4 incorporate additional B16-His and B16-Glu mutations compared to Fc-Ins2. These insulins correspond to compounds 12, 13, and 14 as characterized and reported by Tagmose *et al.*^40^ Based upon *in vitro* potency analysis (Table 1), the additional mutations result in progressively less potent compounds. LY3209590 also contains similar mutations: A14-Asp, B16-Glu, B25-His. These changes have been previously demonstrated to yield insulin analogs with incrementally longer pharmacokinetic profiles. Mean residence times (MRTs) of 43 hours, 60 hours, and 88 hours in STZ-induced Sprague-Dawley rats were reported for Fc-Ins2, Fc-Ins3, and Fc-Ins4, respectively.^40^

**Fig. 2:**
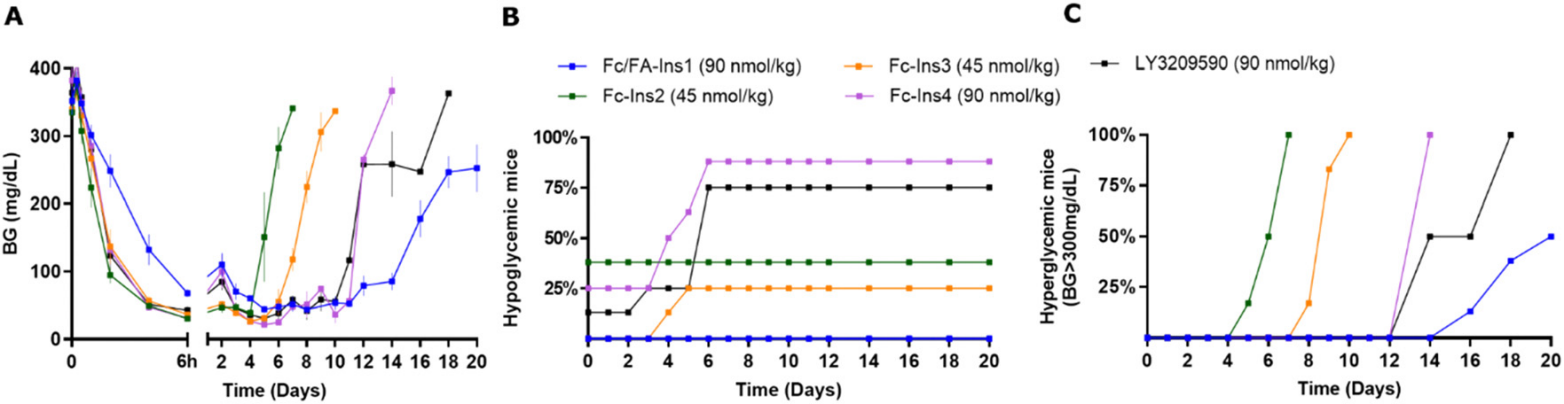
Comparison of PD profiles in STZ-treated mice after a single s.c. dose of previously reported Fc-Ins analogs *vs.* Fc/FA-Ins1. (A) Blood glucose lowering monitered over period of 20 days. Data are expressed as mean ± SEM. (B) Percent of mice in each group that experienced hypoglycemia (viasual signs and/or BG was lower than 30 mg dL^−1^) and had to be removed from study. (C) Percent of mice that had blood glucose levels reach above 300 mg dL^−1^. We used time required to cross 50% mark as measure for the duration of insulin action.

In a direct comparison focused on glucose-lowering in STZ-treated mice (Fig. 2), the dosing for the compounds Fc-Ins2 and Fc-Ins3 was adjusted to 45 nmol kg^−1^ to accommodate their higher potency. Fc/FA-Ins1, Fc-Ins4, and LY3209590 were administered at 90 nmol kg^−1^. The peptides’ performance aligned with their previously reported pharmacokinetic (PK) profiles. Fc-Ins2 sustained reduced glucose levels for approximately 6 days, Fc-Ins3 for 9 days and both Fc-Ins4 and LY3209590 for 13 days. However, all were dramatically less than the 20-day glucose-lowering observed with the doubly modified insulin, FA/Fc-Ins1. Notably, a significant number of mice receiving the Fc-modified insulin without lipid experienced hypoglycemia (hunched posture, lethargy, BG was lower than 30 mg dL^−1^) and subsequently required intervention, or exclusion from the study. This indicates that the administered doses for these shorter-acting peptides were likely more than the maximum tolerable levels in this mouse model.

### Structure activity relationship with respect to insulin

As we observed the insulin backbone can have impact on the PK performance of Fc-protracted analogs, we next evaluated how different peptide sequences and site of conjugation may affect the PK/PD profile of doubly protracted insulins. Fc/FA-Ins5 has protraction moved to an additional non-native lysine at position A22 and Fc/FA-Ins1(B1) is an analogue that is covalently modified at the N-terminus of the B chain. We tested three other backbones which constitute desB30 insulin of native insulin sequence, Ins7 with B16His mutation similar to the analog Ins3, and Ins6 which includes an additional disulfide bond, A10–B3. The reason for selecting these analogs is additional proteolytic stability and improved PK provided by these mutations.^16^ There was considerable drop in *in vitro* potency for the analogs Fc/FA-Ins6 and Fc/FA-Ins7 (Table 1). Reduction in potency and receptor affinity was anticipated based upon the previous results with lipidated analogs.^16^ There was otherwise no substantial difference in potency between B29, B1 and A22 protracted analogs that share a similar insulin backbone.

**Fig. 3:**
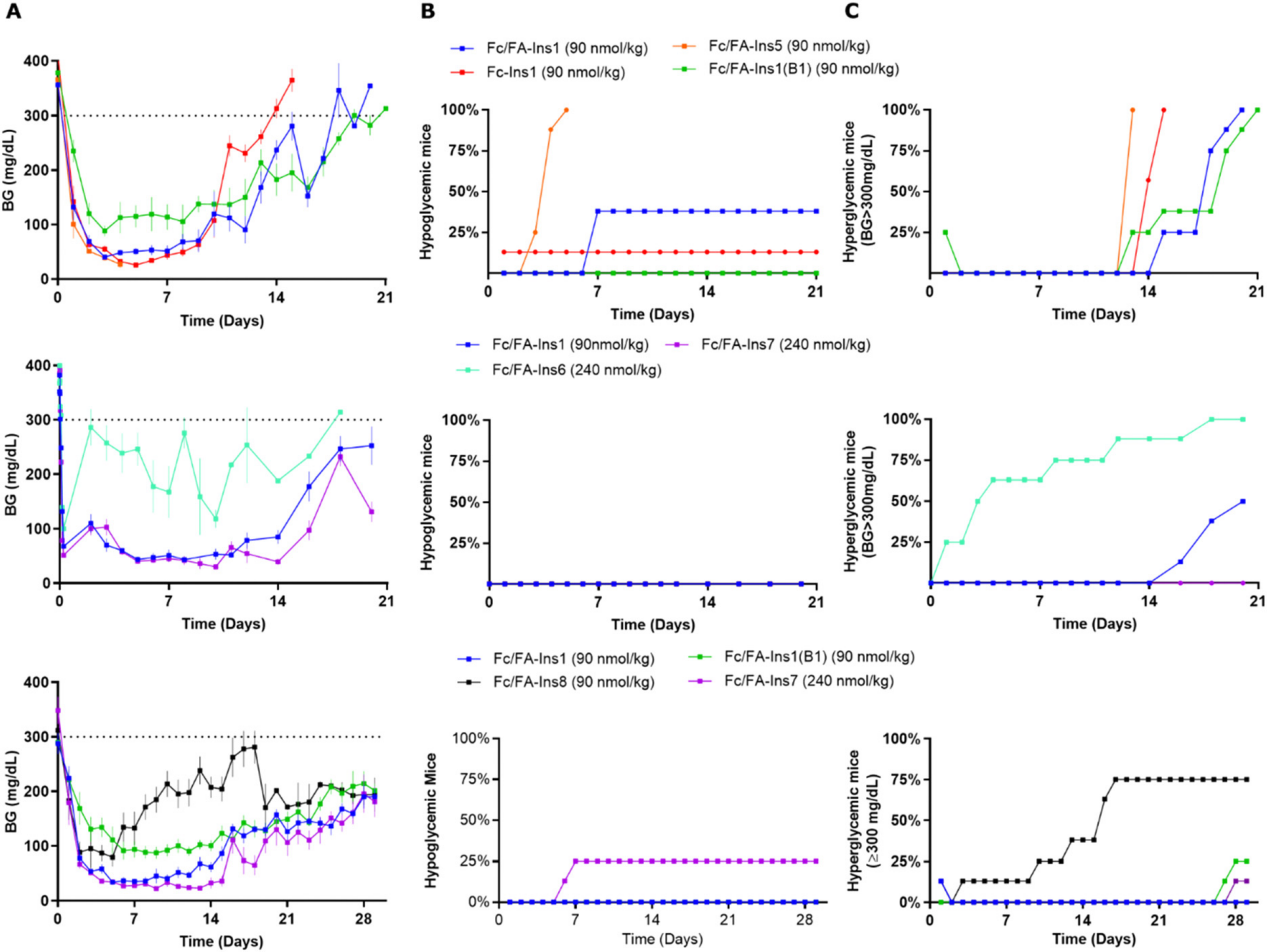
Comparison of PD profiles in STZ-treated mice after a single s.c. dose of Fc/FA-protracted insulins with variations in insulin sequence and protractor attachment point. (Column A) Blood glucose lowering monitored over period of 21 or 30 days. Data are expressed as mean ± SEM. (Column B) Percent of mice in each group that experienced hypoglycemia (viasual signs and/or BG was lower than 30 mg dL^−1^) and had to be removed from study. (Column C) Percent of mice that had blood glucose levels reach above 300 mg dL^−1^. We used time required to cross 50% mark as measure for the duration of insulin action.

As before, we dosed analogs in STZ mice and assessed glucose lowering over a period of 3–4 weeks (Fig. 3). Insulin Fc/FA-Ins1(B1) was tested twice and both times it displayed similarly long duration of action relative to Fc/FA-Ins1. We observed that this analog does not achieve the same level in blood glucose lowering as other insulin analogs and maintains a minimum glucose concentration at ∼100 mg dL^−1^. Insulin Fc/FA-Ins5 (extended from A22) has shown remarkably increased *in vivo* efficacy that is reflected in acute hypoglycemic response in 7 out of 8 animals tested in this study (Fig. 3A, middle panel). No conclusions could be made with the remaining mouse that maintained reduced blood glucose levels for 12 days. Analogs Fc/FA-Ins6 and Fc/FA-Ins7 were dosed at 240 nmol kg^−1^ to compensate for their lower potency. Insulin Fc/FA-Ins7 displayed a similar glucose lowering profile to Fc/FA-Ins1 (Fig. 3B and C), whereas Fc/FA-ins6 was not sufficiently efficacious at this dose and underperformed relative to the other analogs (Fig. 3B). Finally, analog Fc/FA-Ins8 composed of an unmodified desB30 insulin backbone has also proven deficient in its ability to produce sustained glucose lowering in STZ animals.

### Structure activity relationship in the geometry of doubly protracted molecules

The spatial arrangement and proximity of the three components within the Fc/FA-Ins molecule are hypothesized to significantly influence its mechanism of action. To explore this, we synthesized a series of analogs. The (Fc/FA)inv-Ins1 analog reverses the positions of the Fc and the fatty acid, positioning the Fc domain at a greater distance from the insulin moiety, now separated by two long spacers. The variants (Fc/FA)v1-Ins1, (Fc/FA)v2-Ins1, and (Fc/FA)v3-Ins1 have shortened spacers between individual components. The (Fc/FA)half-Ins1 features only a single Fc domain rather than a dimer. These structural modifications did not substantially affect the potency at the insulin receptor, which remained within 1–4% of that of native insulin, as shown in Table 1.

**Fig. 4:**
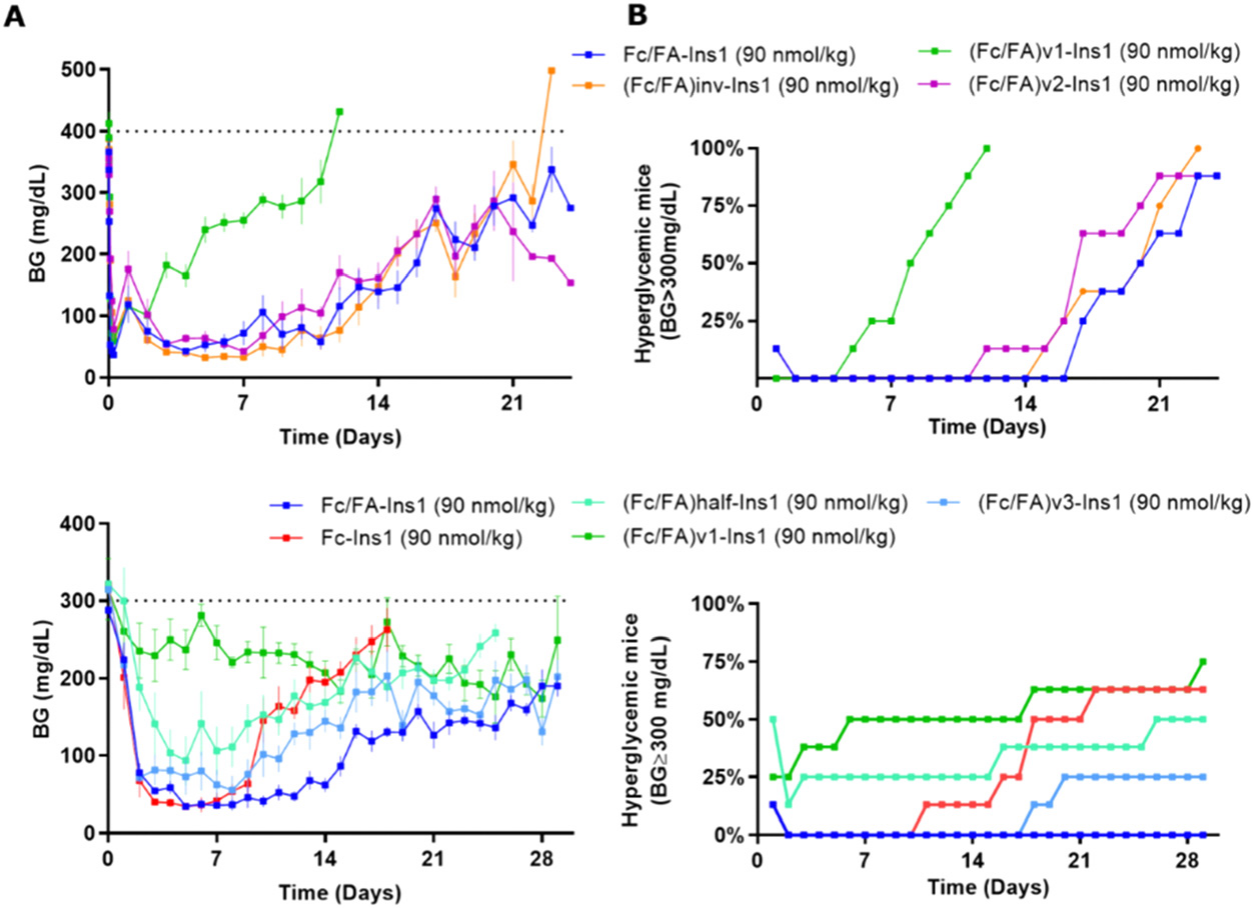
Comparison of PD profiles in STZ-treated mice after a single s.c. dose of Fc/FA-Ins1 variants with changes in the protractor (linker length, relative position of Fc And FA). (Column A) Blood glucose lowering monitered over period of 28 or 30 days. Data are expressed as mean ± SEM. (Column B) Percent of mice that had blood glucose levels reach above 300 mg dL^−1^. We used time required to cross 50% mark as measure for the duration of insulin action.

Experiments in STZ-treated mice indicated that altering the positions of the Fc and FA elements does not significantly impact the insulin analog's efficacy and duration of action (Fig. 4). Similarly, reducing the spacer length between Fc and FA in (Fc/FA)v2-Ins1 and (Fc/FA)v3-Ins1 (shortened to four and two Ado units, respectively) did not affect performance. The notable change occurred with the (Fc/FA)v1-Ins1 analog, where a shorter spacer between insulin and Fc was employed. Although it retained glucose-lowering capabilities, the duration of its action was reduced to around 8 days. Additionally, the removal of one of the Fc domains negatively affected the molecule's overall efficacy and decreased the duration of the glucose-lowering effect.

### Pharmacokinetic study in dogs

To further substantiate the prolonged pharmacokinetic profile of dual-protracted insulin analogs and to confirm that this effect is not exclusive to rodents, we conducted a PK study in dogs. Along with Fc/FA-Ins1 and its comparator Fc-Ins1, which were previously examined in rats, we included two additional molecules with inactive single-chain DesDi insulin analogs. Similar to the design of Ins1 analogs, Fc/FA-Ins9 is a doubly-modified analog with both Fc and fatty acid, while Fc-Ins9 is an Fc-only control. As expected, both were inactive in insulin receptor activation assays. We initially conducted a pilot PK study of the inactive analogs in rats, where animals were dosed at 2 nmol kg^−1^.

An increase in half-life of the inactive molecules was observed relative to their active counterparts (Fig. S3 and Table S2, ESI†). The Fc-only analogs increased from 76 h to 132 h and the Fc/FA analogs from 124 h to 239 h, demonstrating in each instance the importance of receptor-mediated clearance. The dog PK study involved intravenous injection of the four peptides at a dose of 5 nmol kg^−1^ with three animals per group (Fig. S4–S7 and Table S3, ESI†). Plasma samples were collected over two months period and the remaining insulin levels were measured using a luminescent oxygen channelling assay (LOCI) with a pair of antibodies against insulin and Fc. In some subjects, we noted a rapid increase in the clearance rate of insulin analogs at 300–600 hours post-injection, which we attribute to the emergence of anti-drug antibodies (ADAs). Consequently, in Table 3, we present the average PK parameters including data from all animals, as well as a subset excluding ADA-affected outliers. Both datasets indicate a 2- to 3-fold increase in half-life for the dual-protraction compounds compared to Fc-only insulins. The half-life of the active Ins1 analog extended from 78 hours to 226 hours with lipidation, while that of the inactive Ins9 analog increased from 229 hours to 472 hours. As with the rats, there is a noticeable difference between the active and inactive insulin analogs, attributable to clearance through the insulin receptor.^42^

**Table 3:** Pharmacokinetic parameters of active Ins1 and inactive Ins9 insulin analogs from IV administration in dogs

Analyte	Subject	Dose (nmol kg^−1^)	# of animals	HL_Lambda_z (h)	AUC_%Extrap (%)	C0 (pmol L^−1^)	AUCINF (h*pmol L^−1^)	Vz (L kg^−1^)	Cl (L h^−1^ kg^−1^)	Vss (L kg^−1^)
Fc/FA-Ins1	Mean	5	3	226	12	14 3000	2.14 × 10^+07^	0.077	2.35 × 10^−04^	0.0652
	SD			20	19	25 400	2.47 × 10^+06^	0.013	2.56 × 10^−05^	0.0051
	Outliers removed^a^	5	2	230	1.1	131 000	2.01 × 10^+07^	0.083	2.50 × 10^−04^	0.066
	SD			19	0.3	14 000	3.50 × 10^+05^	0.008	4.00 × 10^−06^	0.004
Fc-Ins1	Mean	5	3	78	1.0	101 000	4.69 × 10^+06^	0.125	1.10 × 10^−03^	0.106
	SD			15	1.3	11 400	1.07 × 10^+06^	0.037	2.24 × 10^−04^	0.025
	Outliers removed	5	3	78	1.0	101 333	4.69 × 10^+06^	0.124	1.10 × 10^−03^	0.106
	SD			15	1.3	11 400	1.07 × 10^+06^	0.037	2.24 × 10^−04^	0.025
Fc/FA-Ins9	Mean	5	3	472	33	122 000	4.34 × 10^+07^	0.077	1.33 × 10^−04^	0.0748
	SD			239	14	8870	1.81 × 10^+07^	0.009	6.66 × 10^−05^	0.0078
	Outliers removed	5	1	720	26	127 000	5.97 × 10^+07^	0.087	8.38 × 10^−05^	0.0838
	SD			0	0	0	0.00 × 10^+00^	0	0.00 × 10^+00^	0
Fc-Ins9	Mean	5	3	229	6.3	65 900	8.90 × 10^+06^	0.187	5.69 × 10^−04^	0.177
	SD			33	7.5	10 900	1.23 × 10^+06^	0.024	7.58 × 10^−05^	0.022
	Outliers removed	5	1	256	2.5	56 600	8.62 × 10^+06^	0.214	5.80 × 10^−04^	0.203
	SD			0	0	0	0.00 × 10^+00^	0	0.00 × 10^+00^	0

a Two sets of values are reported: one with all animals included (highlighted in bold) and another where we removed outliers associated with development of anti-drug antibodies (graphs and detailed data reported in ESI).

### Maintenance dosing regimen in STZ mice

**Fig. 5:**
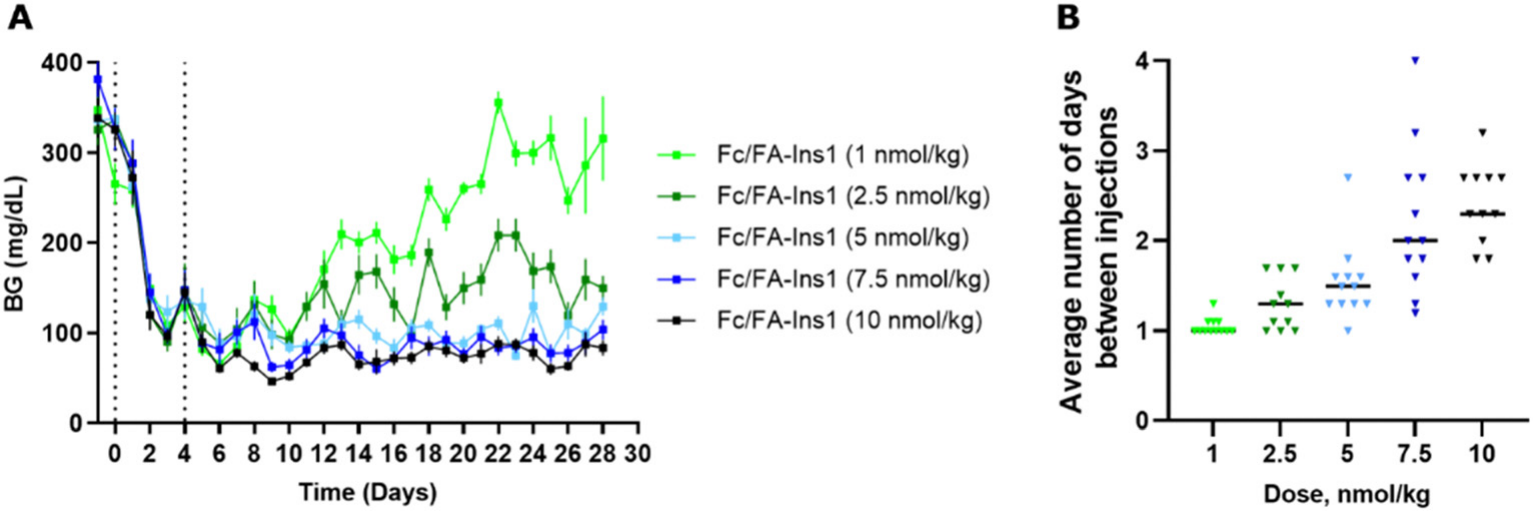
Blood glucose maintenance experiment to determine frequency of injections required to keep glucose levels at 75 mg dL^−1^. (A) Blood glucose levels in STZ mice which were conditionally dosed with low-dose of Fc/FA-Ins1 over 4 weeks. After initial injection of 50 nmol kg^−1^ of insulin and 4 days initiation period, animals were placed on maintenance regimen and insulin was administered to each mouse that has blood glucose reading 75 mg dL^−1^ or above. Data are expressed as mean ± SEM. (B) Frequency of injections at each dose for individual animals in the study and the average dosing frequency.

To better reflect a clinical setting where insulin is repeatedly administered at lower doses, we simulated such conditions in a rodent study. We implemented an insulin dosing regimen where Fc/FA-Ins1 was administered to animals only when their blood glucose levels exceeded a predetermined threshold. Initially, we reduced blood glucose by administering a 50 nmol kg^−1^ dose of Fc/FA-Ins1 to the animals (see Fig. 5). On day 4, we commenced the maintenance phase, employing five different doses ranging from 1 to 10 nmol kg^−1^. This phase spanned over 4 weeks or until animals had to be withdrawn from the study due to high blood glucose levels (exceeding 300 mg dL^−1^). Animals received doses only when glucose levels rose above 75 mg dL^−1^.

In the group receiving the smallest maintenance dose of 1 nmol kg^−1^, animals required daily dosing, and blood glucose levels could not be effectively controlled, leading to eventual hyperglycemia in all animals. At a slightly higher dose of 2.5 nmol kg^−1^, the dosing frequency decreased, and blood glucose was generally maintained between 100–200 mg dL^−1^ throughout the study. For the remaining three dosing groups—5 nmol kg^−1^, 7.5 nmol kg^−1^, and 10 nmol kg^−1^—the average dosing intervals were approximately every 1.5 days, 2 days, and 2.3 days, respectively, with all groups successfully maintaining blood glucose levels below 100 mg dL^−1^. Based on these results, a maintenance dose of ∼4 nmol kg^−1^ is necessary as a daily insulin treatment in STZ-treated mice.

## Discussion

### Consideration of long-acting insulin design

While the protraction mechanism is intriguing, it is essential to consider individual elements in the molecular design. Even with a pharmacokinetic extender, the performance of the molecule will be governed by rate-limiting factors. One such critical element is the proteolytic stability of the insulin backbone itself. We have drawn extensively from previous research to ensure that the insulins used in this study are optimized for *in vivo* stability.^41^ Mutations like A14-Glu and B25-His provide the necessary protection against serum proteases and enhance the overall biophysical stability of the insulin molecule. Additionally, reduced *in vitro* potency is a significant characteristic for long-acting insulin variants, as it decelerates receptor-mediated clearance to provide increased effective insulin dose, which is required for prolonged efficacy.

Separating the contributions of potency and stability to the overall efficacy of an insulin analog may be challenging in our case, but their significance is underscored when comparing Fc/FA-Ins8 and Fc/FA-Ins9. The former has the native insulin backbone, which has higher potency and lower proteolytic stability, and showed reduced *in vivo* efficacy compared to analogs with double mutations. The latter, being an inactive single-chain insulin analog, displayed a notable increase in half-life—doubling that seen in rat and dog models compared to Ins1 molecules. Although Fc/FA-Ins9 lacks the specific mutations that typically enhance stability, the improved stability could potentially stem from the compact single-chain structure of the DesDi insulin analog.

Achieving a balance between stability and potency is crucial for the development of insulin analogs. While the additional stabilizing mutations in Fc/FA-Ins6 and Fc/FA-Ins7 could potentially yield superior analogs in terms of stability, this benefit may come at the expense of increased dosage requirements. Fc/FA-Ins7 achieves a similar level of efficacy to Fc/FA-Ins1 but necessitates a 2.5-fold higher dose. In this context, the B16-His mutation may be considered disadvantageous. However, it seems to confer a longer duration of action, as no hypoglycemia was observed at end of the experiment on day 21 (Fig. 3B), unlike the Fc/FA-Ins1 group where half of the animals reached hypoglycemia at that point. The 4-disulfide insulin structure of Fc/FA-Ins6 further diminished potency, suggesting that an even higher dose would be required to observe a comparable *in vivo* efficacy.

Finally, the site at which insulin is extended can significantly influence its pharmacokinetic properties. For the Fc/FA-Ins1(B1) analog, a notable difference is the plateau concentration in blood glucose that is achieved in the study. This plateau is higher than those observed with the other insulin analogs tested and was maintained at approximately 100 mg dL^−1^. If these findings are both reproducible and translatable, it implies that insulin analogs protracted at the B1 position might result in a safer glucose-lowering profile that minimizes the risk of hypoglycemia. In contrast, the Fc/FA-Ins1(A22) analog demonstrates greater *in vivo* activity, as evidenced by the increased incidence of hypoglycemic events observed in our study.

### Mechanistic considerations

The finding that the distance between the Fc and the lipid had no profound effect on the duration of action was surprising to us. We initially hypothesized that the Fc domain and the lipid-albumin complex would both interact with the neonatal Fc receptor (FcRn) within the endosome, to achieve increased affinity and enhanced recycling of the resultant quaternary complex (as depicted in Fig. S1, ESI†). The distance between the Fc and lipid moieties was thus needed to span the gap between Fc and albumin to facilitate this complex formation. However, the evidence suggests otherwise, and an alternative hypothesis must be considered.

One possibility is that two distinct FcRn receptors are engaged concurrently—one *via* the Fc portion and the other *via* the fatty acid moiety when bound to albumin. The distance in this case only needs to be sufficient to avoid steric clash between the two FcRn receptors. Another hypothesis could involve direct interactions of the fatty acid with the receptor or the phospholipid membrane. Additionally, the mere dual presence of FcRn-binding elements might increase the probability of interaction and subsequent FcRn-mediated recycling. Finally, the extension in pharmacokinetics could simply be due to the binding to plasma albumin. A more detailed investigation will be necessary to accurately determine the mechanism behind the observed effects.

Another surprising observation was that the distance between the insulin and Fc domains appears to be a significant factor. Although we did not detect any impact of a shorter linker on the potency at the insulin receptor, it is conceivable that the proximity of insulin to the Fc domain might influence the binding to the neonatal Fc receptor (FcRn), potentially disrupting the recycling process and leading to poorer pharmacokinetic (PK) characteristics.

Both insulin and Fc interact with their respective receptors in a pH-dependent manner. The acidic environment of the endosome promotes the dissociation of insulin from the insulin receptor. Conversely, Fc binding is enhanced at the lower pH of 5.5, which is conducive to the effective recycling of Fc. It is plausible to consider a 'hand-over' from one receptor to another takes place, which would necessitate the capacity for simultaneous binding to both receptors and adequate spacing between the two ligands. Once again, to validate these hypotheses and elucidate the precise mechanism, a more comprehensive mechanistic investigation is required.

### Wider implications

The immediate implication of this work is the potential to develop ultra-long-acting insulin analogs through the combined use of Fc and fatty acid protraction strategies. This creates an opportunity to produce analogs that require less frequent dosing, potentially paving the way for a once-monthly basal insulin regimen. The pharmacokinetic data from the canine study seem to support this possibility. While applying allometric scaling to such constructs may be challenging, the collective results from rat and dog studies suggest that the half-life of doubly protracted insulin will double in comparison to Fc-only analogs. Taking into consideration PK results from LY3209590, which displays a half-life in rats of ∼120 h and in human ∼17 days, we can hypothesize such insulin analogs may reach half-life of ∼30 days.^38,43^

Alternatively, such insulin analogs could be administered more frequently (once weekly) to establish an exceptionally stable basal insulin profile by providing a consistent steady-state concentration of insulin over an extended period. The absence of hypoglycemic events with the FA/Fc-Ins1 analog might suggest an additional margin of safety for compounds with dual protraction mechanisms, which could extend beyond simply providing a flat exposure profile. However, this potential safety benefit would need to be substantiated with further dose-escalation safety studies. An additional protective factor against hypoglycemia is the slower initial decrease in blood glucose levels observed within the first 6 hours post-injection (Fig. 1), which helps to guard against hypoglycemia that could potentially be triggered by the injection itself.

Another safety feature may arise from the increased hydrodynamic size of the Fc/FA-Ins molecule. The molecular weight of the molecule itself is approximately 50 kDa, which is expected to increase to around 120 kDa upon binding to albumin. This larger size will restrict the molecule's exposure to peripheral tissues, favoring a more liver-targeted (hepato-preferential) action profile. Such hepato-preferential effects have been observed individually with both Fc-modified and lipidated insulin analogs.^44,45^ This opens intriguing possibilities for synergy that could results from the combination of these two modifications.

The extended pharmacokinetic (PK) profile of insulin analogs can be a double-edged sword. While the benefits have been listed above, a significant concern with this type of profile is the risk associated with potential overdose and hypoglycemia.^46,47^ Counteracting such a long-acting insulin with glucose might prove to be extremely difficult and would require intensive and persistent medical intervention. Considering this, additional safety mechanisms could be considered. One such measure might involve engineering a specific cleavage site between the insulin and Fc components, which could be targeted and selectively cleaved by an exogenously administered protease. Another strategy could be the development of anti-insulin antibodies that are tailored to selectively bind to the unique insulin mutants employed in the construction of these long-acting analogs. Finally, targeted protein degradation techniques could be harnessed to selectively eliminate any excess insulin present in the system.^48^ Such approaches could provide a means to mitigate the risks associated with the prolonged activity of insulin analogs.

Another concern comes from the dog study where we observe the development of anti-drug antibodies (ADAs).^49,50^ However, given that both the insulin and Fc components are based on human sequences, it is uncertain whether a similar immune response will occur in human subjects. Efsitora alfa that incorporates human Fc protein in its design did not show any issues with ADA formation in clinical studies. The analogs reported in this study are certainly not optimized for clinical application, and further structural refinements may be necessary while taking this concern into consideration.

Fundamentally, the strategy of combining Fc domain with fatty acid modification has implications that extend beyond insulin, offering potential applications for a variety of other peptide and protein therapeutics. At least one report supports the notion that the approach is expandable to other peptides.^51^ Our decision to investigate inactive insulin analogs was, in part, to explore the maximal extent of this protraction strategy. By doing so, we aimed to delineate the effects of insulin-mediated activity and clearance from those solely attributable to the pharmacokinetic-enhancing capabilities of the Fc/FA construct. As anticipated, the exclusion of active clearance mechanisms through the insulin receptor markedly increased the circulating half-life of both Fc-conjugated and Fc/FA-conjugated molecules. In our studies, the Fc-only construct achieved a half-life of 132 hours (5.5 days) in rats and 256 hours (10.7 days) in dogs. The Fc/FA-conjugated analog demonstrated an even more prolonged half-life of 238 hours (10 days) in rats and extended up to 720 hours (30 days) in dogs.

## Conclusions

This study showcases how a combination of two well-established strategies for prolonging the pharmacokinetic profile of therapeutics can lead to a synergistic outcome. Our work is centered on insulin therapy by developing analogs that can maintain glucose-lowering activity for up to two to three weeks in STZ-induced diabetic mice. Should this be translatable to humans, it may result in the creation of a basal insulin capable of achieving a once-monthly dosing schedule. Importantly, such an insulin would offer a more consistent steady-state concentration, potentially mitigating the risks associated with fluctuations in insulin activity. The extended duration of action characteristic of such insulin analogs is not without its challenges, and we foresee that these could limit the feasibility of the therapy. Crucially, the principles underlying this approach hold promise for a wider array of peptide- and protein-based treatments, potentially ushering of a new class of long-acting therapeutic agents.

## Materials and methods

### Peptide synthesis

Linkers were synthesized with an Applied Biosystems 433A Peptide Synthesizer using preprogrammed solid phase Fmoc protocol designed for 0.1-mmol syntheses. DIC/HOBt-Cl in NMP were used for coupling and 20% piperidine in NMP for deprotection. Insulin peptides were assembled on Symphony X instrument (Gyros Protein Technologies) using 30-minute coupling cycles with 0.3 M AA activated by DIC/Oxyma and 20% piperidine in DMF for deprotection. Peptides were cleaved with TFA cocktail (15 mL per 0.1 mmol of resin) containing 2.5% v/v of each: β-mercaptoethanol, triisopropylsilane, anisole and water. Insulins were folded by first dissolving crude peptide in 200 mL 100 mM Na_2_CO_3_ buffer, pH11 with 100 mg Cys-HCl. 500 mg of cystine was dissolved in 1 N NaOH and added to the stirring peptide solution. After 20 minutes, solution was acidified using TFA and folded peptide purified by preparative HPLC. Lys-C cleavage was done by adding 0.01 equiv. of enzyme (produced internally at NovoNordisk) to purified insulin peptide dissolved in 20% MeCN–50 mM NH_4_HCO_3_ buffer, pH 8. Final insulin purified by prep HPLC.

### Analysis and purification

Analytical HPLC was performed on an Agilent's 1260 Infinity/6120 Quadrupole instrument with Kinetex C8 2.6m 100A (75V60 mm) column using a flow rate of 1 mL min^−1^ and a gradient 10–80% B over 10 min. Eluent A is water with 0.05% TFA and eluent B is 10% water, 90% MeCN, 0.05% TFA. MS collected in 200–2000 *m*/*z* range. HPLC purifications were performed on a Waters instrument (Waters Controller model 600, Waters dual wavelength detector 2487, ProStar model 701 fraction collector and Kipp & Zonen BD41 chart recorder) with Luna 10m C8(2) 100A AXIA (250V21.2 mm) column and flow rate of 10–15 mL min^−1^. Protein purification was performed on ÄKTA Pure Protein Purification System using ion-exchange Source 15Q media (Cytiva), with salt gradient 0 to 0.15 M NaCl in 10 mM phosphate buffer, pH 7.4. All compounds are >95% pure by HPLC analysis.

### Fc/FA-Ins synthesis

Linker was assembled on 2-chlorotrityl resin (0.3 mmol, 1.1 mmol g^−1^ loading) preloaded with Fmoc-AEEA-OH (Fmoc-Ado-OH) on ABI433 peptide synthesizer. Fmoc-Lys(ivDde)-OH was used at the branching position and C20-diacid mono-*t*-butyl ester on the N-terminal end. Resin was treated with 2% hydrazine in DMF for 2 hours to remove ivDde protecting group. Free amine was then coupled with Fmoc-Lys(Fmoc)-OH, followed by piperidine deprotection and coupling with iodoacetic acid. Linker was cleaved from the resin in 15 mL THF containing 1% TFA for 3 hours; the crude was diluted into 30% MeCN/0.1% TFA aqueous buffer and lyophilized. C-terminal carboxylic acid was converted to NHS ester by reaction with *N*-hydroxysuccinimide/DIC (1 mmol) in THF (30 mL) for 2 hours. Crude reaction mixture was diluted with 30% MeCN/0.1% TFA aqueous buffer, purified by prep HPLC and fractions containing NHS ester were immediately lyophilized. To remove *t*Bu protecting groups NHS ester was treated with 2 mL TFA for 1 hour. TFA was removed by rotavap, and residue dried under vacuum for 1 hour. NHS ester was dissolved in MeCN and slowly added to insulin (50 mg ml^−1^) dissolved in 100 mM Na_2_CO_3_ buffer, pH 11. pH was maintained above 10.5 to ensure selective modification of ε-amine on lysine. After 5 minutes product was purified by HPLC and lyophilized. For conjugation to Fc, Fc dimer was first reduced using substoichiometric amount of TCEP in 50 mM Tris buffer, pH 8. Insulin was dissolved in small volume of 30% MeCN/0.1% TFA aqueous buffer and combined with reduced Fc. pH of the reaction was readjusted to 8.0 and completion was monitored by LCMS. Fc conjugate was purified by ion-exchange chromatography and clean fractions were concentrated using 5 kDa MWCO spin columns.

### Fc-Ins synthesis

Linker was assembled on 2-chlorotrityl resin (0.3 mmol, 1.1 mmol g^−1^ loading) preloaded with Fmoc-AEEA-OH (Fmoc-Ado-OH) on ABI433 peptide synthesizer. Fmoc-Lys(Fmoc)-OH was placed on the N-terminal position of the linker. After 1 h piperidine deprotection, two iodoacetic acid moieties were coupled using DIC reagent. The linker was cleaved using neat TFA (3 mL) and precipitated in ether (45 mL). After three ether washes, the linker was dissolved 30% MeCN/0.1% TFA aqueous buffer and lyophilized. The procedure for NHS ester synthesis (without *t*Bu deprotection), insulin acylation and conjugation to Fc is identical to Fc/FA-Ins example.

### Insulin receptor *in vitro* phosphorylation assay

Insulin receptor phosphorylation was measured in an indirect ELISA assay. HEK293 cells (ATCC CRL-1573) overexpressing B-isoform of human insulin receptor (hIR-B) were plated in 96-well tissue culture plates and cultured in DMEM supplemented with 100 IU mL^−1^ penicillin, 100 μg mL^−1^ streptomycin, 10 mM HEPES, and 0.25% bovine growth serum (HyClone SH30541) for 16–20 h at 37 °C, 5% CO_2_, and 90% humidity. Serial dilutions of biosynthetic human insulin and test peptides prepared in DMEM supplemented with 1% ovalbumin (Millipore-Sigma, A5503) were added to the plate wells. After 15 min incubation at 37 °C in humidified atmosphere with 5% CO_2_, the cells were fixed with 5% paraformaldehyde for 20 min at room temperature, washed twice with phosphate buffered saline (PBS, pH 7.4) and blocked with 2% bovine serum albumin (BSA, Millipore-Sigma 810533) in PBS for 1 h. The plate was washed three times, filled with anti-phospho-IR/IGF1R (Tyr1158) antibody (Life Technologies, 710101) and incubated for 3 h at room temperature, after which the plate was washed four times and filled with goat anti-rabbit secondary antibody (#A16110, ThermoFisher). Following 30 min incubation and another four washes, 0.1 mL of TMB One Solution substrate (Life Technologies, #00-2023) was added to each well. Color development was stopped 10 min later by adding 0.05 mL 1 M hydrochloric acid. Absorbance at 450 nm was measured on envision multimode reader (PerkinElmer). Absorbance *vs.* peptide concentration dose–response curves were plotted, and EC50 values were determined using logistic nonlinear three-parameter regression in GraphPad Prism 8 (GraphPad Software). Each experiment was performed at least three times, and the error bars represent standard deviation. Dose–response curves can be found in Fig. S8 (ESI†).

### *In vivo* PD studies

All rodent studies were approved by the Institutional Animal Care and Use Committee of the University of Cincinnati in accordance with the US National Institutes of Health Guide for the Care and Use of Laboratory Animals. Mice and rats were housed in an AAALAC-approved room with a 12-h light, 12-h dark cycle room held at 22 °C and with free access to food and water.

### STZ-treated lean mice

C57Bl/6J male mice (Cat. Number #000664, Jackson) were maintained on pelleted chow diet (Teklad LM-485, Envigo; 3.1 kcal g^−1^) and fasted overnight before receiving an injection of streptozotocin (STZ, Sigma Aldrich #S0130, 150 mg kg^−1^, ip) freshly dissolved in ice cold sodium Citrate buffer (pH = 4.5), within the first two hours following the onset of the light phase. The food was returned after the STZ injection and a 10% sucrose solution was also offered for the first 72 hours. Mice exhibiting glycemia lower than 300 mg dL^−1^ 72 hours following STZ administration were excluded from the studies. The hyperglycemic mice received daily injections of 20 kDa-polyethylene glycol-insulin (PEG-INS, 30–40 nmol per kg, s.c.) within 3–4 hours of the onset of the dark phase to maintain normoglycemia. PEG-INS was withdrawn for 72 hours prior the start of the studies.

For acute single-injection studies, mice were fasted at the onset of the light phase for two hours before the first sc injection at *T* = 0. Blood glucose was followed with a handheld glucometer for 6 hours. Mice were refed after the final time point and BG was followed daily within the first two hours of the onset of the light phase as needed. In cases when insulin-treated mice exhibited symptoms of hypoglycemia (*e.g.* hunched posture, lethargy) and/or BG was lower than 30 mg dL^−1^. Those mice received an ip bolus (200 μl) of 20% d-glucose and were euthanized if their condition did not improve within 1 hour.

For chronic studies, all mice were given a sc dose of Fc/FA-Ins1 following the day 0 acute study design detailed above. After 4 days, mice were randomized into groups (*n* = 12) and began to receive maintenance sc injections based on their daily blood glucose, as dictated by the parameters of the study.

### SD rats PD

Sprague-Dawley male rats (280–400 g, Envigo, IN). were maintained on pelleted chow diet (Teklad LM-485, Envigo; 3.1 kcal g^−1^). Rats were fasted at the onset of the light phase for two hours before the first sc injection at *T* = 0. Blood glucose was followed with a handheld glucometer for 6 hours. Rats were refed after the final time point and BG was followed daily within the first two hours of the onset of the light phase as needed.

### SD rats PK (i.v. and s.c.)

PK studies in SD rats were conducted at Novo Nordisk A/S in Denmark and were performed according to guidelines and permissions granted by the Animal Experiments Inspectorate, Danish Ministry of Environment and Food of Denmark or the Departmental Expert Commission on Approval of Animal Experiment Projects. Sprague-Dawley male rats (240–260 g, Janvier, France) were acclimated one week in-house prior to the experiment and were kept in group cages with 3–5 rats in each. During the acclimatization and study period the animals are housed in groups according to Animal Unit's SOP (Housing of experimental animals at Novo Nordisk A/S). The rats are given free access to food (Altromin 1324) and water. Animals are housed in a 12 h light/dark cycle with lights on at 6 am. The rats are weighed the day before or on the day of the experiment (approximately 300 g). Rats had access to food and access to water during the experiment. On day of experiment, animals were brought into the laboratory and acclimated to the room for 30 minutes. Blood samples were taken from the tail vein for BG measurement prior to i.v. and s.c dosing. At time = 0 min, animals will be dosed by i.v. injection in the tail vein (1 ml kg^−1^) or by s.c in the neck (0.5 ml kg^−1^) using Novopen. 5 rats are included in each group. Blood samples are collected from the sublingual plexus for determination of both BG and exposure (1 h, 2 h, 4 h, 6 h, 24 h, 2 d, 3 d, 4 d, 5 d, 8 d, 9 d, 10 d, 11 d, 12 d). No effect on BG was observed at the doses chosen for experiments.

### Dog PK

The dog study protocol used in this study was approved by the institutional animal care and use committee of Novo Nordisk (Denmark), in accordance with Sinclair Research (USA) standard operating procedures. Adult female Beagle dogs of at least 1 year of age and at least 6 kg body weight were randomized based on body weight into groups of three. *Ad libitum* fed dogs were administered a single dose of the insulin analogs *via* a bolus I.V. *via* an in-dwelling catheter at time = 0 with a dose volume of 2.5 ml kg^−1^ body weight. Whole blood (2 mL) was collected *via* direct venipuncture of the jugular vein at time = 0, 1, 1.5, 2, 3, 6, 8, 12, 24, 48, 72, 96, 120, 144, 168, 240, 336, 504, 672, 840, 1008, 1176, and 1344 hours post dose. A small portion of the whole blood was sampled prior to collection for blood glucose measurement using AlphaTRAK 2 system. Blood samples were collected into K2EDTA coated tubes prior to centrifuging for plasma collection for analyte quantification. Dogs were regularly observed and subsequently documented for any clinical signs of illness or reaction to the insulin treatments with a particular emphasis on signs related to hypoglycemia, including pallor/coldness, dilated pupils, weakness, lethargy, vomiting, seizure, abnormal breathing and unconsciousness.

### Plasma samples bioanalysis

Quantitative determination of insulin plasma concentrations was obtained using Luminescence Oxygen Channeling Immunoassay (LOCI)/Alpha-LISA^52,53^ and suitable antibodies against the Fc and insulin parts of the molecule as previously described.^40^ Briefly, donor beads (Alpha-LISA donor beads, PerkinElmer, Waltham, Massachusetts, USA) were coated with streptavidin, while acceptor beads (Alpha-LISA acceptor beads, PerkinElmer, Waltham, Massachusetts, USA) were conjugated with a mAb specific for the Insulin part of the module. A second pAb, recognizing the Fc part, was biotinylated. The three reactants were combined with the compound of interest to form a two-sited immuno-complex. Illumination of the complex resulted in a chemiluminescence response which was measured in an Envision plate reader (PerkinElmer, Waltham, Massachusetts, USA). The amount of light was proportional to the concentration of the analyte. Quantification of FA-Ins1 in plasma was obtained using LOCI and two mAbs against insulin.

The pharmacokinetic parameters of the protracted insulin analogues were calculated by non-compartmental analysis using Phoenix WinNonlin version 8.1 (Certara, Princeton, NJ, USA).

## Author contributions

A. N. Z. conceptualization, experimental design, synthesis and characterization, writing; V. M. G. *in vitro* assay; T. M. T. synthesis, editing; D. D. rat PK, V. M. bioanalysis; R. R. PK analysis; M. R. *in vivo* rodent studies; D. P. T. supervision, *in vivo* experiment design; B. F. PD and PK study design, editing; R. D. D. conceptualization, supervision, editing.

## Data availability

The data supporting this article have been included as part of the ESI.†

## Conflicts of interest

There are no conflicts to declare.

## Supplementary Material

CB-005-D4CB00078A-s001
