# Supplementary material for: Toward once-monthly insulin therapy via synergy in two pharmacokinetic protractors: Fc-conjugation and fatty acid acylation
Source: RSC Chem Biol. 2024 Jun 18;5(8):763–75. doi: 10.1039/d4cb00078a (PMC11289878; doi:10.1039/d4cb00078a)
Supplement: CB-005-D4CB00078A-s001 [file CB-005-D4CB00078A-s001.pdf]

Electronic Supplementary Material (ESI) for RSC Chemical Biology.
This journal is © The Royal Society of Chemistry 2024

# Supporting Information

# Toward once-monthly insulin therapy via synergy in two pharmacokinetic protractors: Fc-conjugation and fatty acid acylation.

Alexander N. Zaykov,[a] Vasily M. Gelfanov,[a] Tina M. Tagmose,[b] Damien Demozey,[b] Valentina Manfè,[b] Rebecca Rohlfs,[a] Marita Rivir,[c] Diego Perez-Tilve,[c] Brian Finan,[a] and Richard D. DiMarchi [a],[d]

[a.] *Novo Nordisk Research Center Indianapolis, Indianapolis, IN, 46241, USA*
[b.] *Novo Nordisk, Global Research Technologies, DK-2760 Maaloev, Denmark*
[c.] *Department of Pharmacology and Systems Physiology, University of Cincinnati-College of Medicine, Cincinnati, OH, 45267, USA*
[d.] *Department of Chemistry, Indiana University, Bloomington, IN, 47405, USA*
*† Corresponding Author: Alexander N. Zaykov – Novo Nordisk Research Center Indianapolis, Indianapolis, IN, 46241, USA. E-mail:* ***alexander.n.zaykov@gmail.com***

## Table of content

Figure S1. Human albumin and Fc in complex with FcRn receptor (PDB 4N0U). S2

Figure S2. Pharmacokinetic profiles compounds Fc/FA-Ins1, Fc-Ins1, Fc/FA-Ins1(B1) and FA-Ins1 from s.c. PK study in rats. S2

Table S1. PK parameters for compounds Fc/FA-Ins1, Fc-Ins1, Fc/FA-Ins1(B1) and FA-Ins1 from s.c. PK study in rats. S2

Figure S3. Pharmacokinetic profiles of compounds Fc-Ins9, Fc/FA-Ins6, Fc/FA-Ins7, and Fc/FA-Ins9 from i.v. PK study in rats. S3

Table S2. PK parameters of compounds Fc-Ins9, Fc/FA-Ins6, Fc/FA-Ins7, and Fc/FA-Ins9 from i.v. PK study in rats. S3

Figure S4. Pharmacokinetic profiles from individual animals in dog PK study, Fc/FA-Ins1 S4

Figure S5. Pharmacokinetic profiles from individual animals in dog PK study, Fc-Ins1 S4

Figure S6. Pharmacokinetic profiles from individual animals in dog PK study, Fc/FA-Ins9 S4

Figure S7. Pharmacokinetic profiles from individual animals in dog PK study, Fc-Ins9 S4

Table S3. Pharmacokinetic parameters from individual animals in dog PK study S5

Figure S8. Representative dose-response curves from in-vitro insulin receptor activity assays. S6-S7

Figure S9. A generalized synthetic scheme for preparation of Fc/FA-Ins1. S8

Table S4. MS characterization summary of the insulin compounds prepared in this study. S9

Structures and LCMS data of the insulin compounds prepared in this study. S9-S25

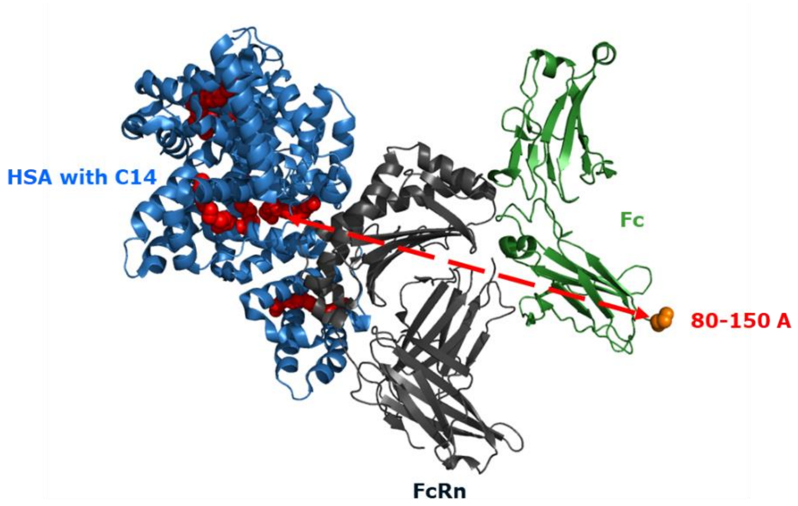


**Figure S1.** Human albumin and Fc in complex with FcRn receptor (PDB 4N0U). The distance from fatty acids binding sites to the N-terminus of Fc protein varies from 80 to 150A. We hypothesized that a spacer with 10 Ado (OEG) units, which approximately 110A in length, will allow simultaneous binding of albumin and Fc, and formation of quasi-quaternary complex.

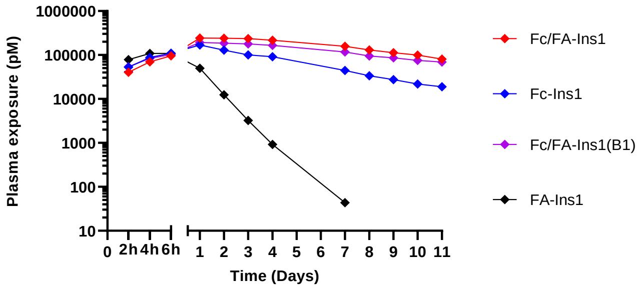


**Figure S2.** Pharmacokinetic profiles compounds Fc/FA-Ins1, Fc-Ins1, Fc/FA-Ins1(B1) and FA-Ins1 from s.c. PK study in rats.

| Compound | Stat | Dose (nmol) | # of animals | HL_Lambda_z (hr) | Cmax (pmol/L) | AUCINF_pred (hr*pmol/L) | AUC_%Extrap_pred (%) | Vz_pred (L/kg) | Cl_pred (L/hr/kg) |
|---|---|---|---|---|---|---|---|---|---|
| **Fc-Ins1** | Mean | 30 | 5 | 124 | 2.52E+05 | 6.09E+07 | 26 | 0.087 | 4.97E-04 |
| | SD | | | 29 | 1.48E+04 | 6.14E+06 | 7 | 0.013 | 5.26E-05 |
| **Fc/FA-Ins1** | Mean | 30 | 5 | 76 | 1.69E+05 | 2.12E+07 | 9 | 0.155 | 1.42E-03 |
| | SD | | | 6 | 1.68E+04 | 1.28E+06 | 2 | 0.009 | 9.10E-05 |
| **FA-Ins1** | Mean | 30 | 5 | 135 | 1.98E+05 | 4.79E+07 | 28 | 0.122 | 6.29E-04 |
| | SD | | | 16 | 1.39E+04 | 3.87E+06 | 4 | 0.007 | 5.07E-05 |
| **Fc/FA-Ins1(B1)** | Mean | 30 | 5 | 13 | 1.11E+05 | 2.92E+06 | 0.3 | 0.188 | 1.03E-02 |
| | SD | | | 1 | 1.26E+04 | 1.51E+05 | 0.2 | 0.021 | 5.58E-04 |

**Table S1.** PK parameters for compounds Fc/FA-Ins1, Fc-Ins1, Fc/FA-Ins1(B1) and FA-Ins1 from s.c. PK study in rats.

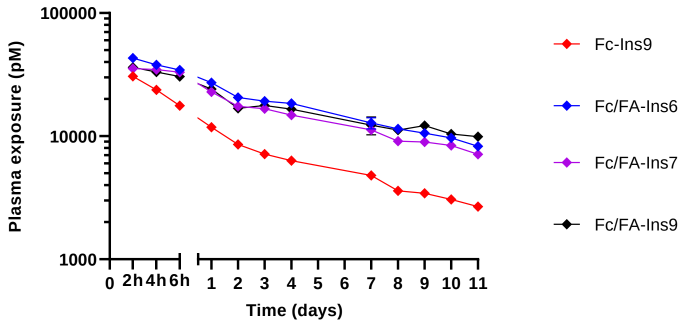


**Figure S3.** Pharmacokinetic profiles of compounds Fc-Ins9, Fc/FA-Ins6, Fc/FA-Ins7, and Fc/FA-Ins9 from i.v. PK study in rats.

| Compound | Stat | Dose (nmol/kg) | # of animals | HL_Lambda_z (hr) | Cmax (pmol/L) | AUCINF_pred (hr*pmol/L) | AUC_%Extrap _pred (%) | Vz_pred (L/kg) | Cl_pred (L/hr/kg) |
|---|---|---|---|---|---|---|---|---|---|
| **Fc-Ins9** | Mean | 2 | 5 | 132 | 30620 | 2.26E+06 | 23 | 0.169 | 8.88E-04 |
| | SD | | | 4 | 2520 | 1.64E+05 | 2 | 0.011 | 6.16E-05 |
| **Fc/FA-Ins9** | Mean | 2 | 5 | 239 | 36460 | 7.58E+06 | 44 | 0.09 | 2.65E-04 |
| | SD | | | 64 | 2971 | 6.16E+05 | 7 | 0.016 | 3.07E-05 |
| **Fc/FA-Ins6** | Mean | 2 | 5 | 153 | 43300 | 6.29E+06 | 29 | 0.071 | 3.21E-04 |
| | SD | | | 8 | 1900 | 6.36E+05 | 2 | 0.008 | 2.33E-05 |
| **Fc/FA-Ins7** | Mean | 2 | 5 | 164 | 30620 | 5.39E+06 | 32 | 0.088 | 3.72E-04 |
| | SD | | | 19 | 2520 | 3.64E+05 | 5 | 0.012 | 2.01E-05 |

**Table S2.** PK parameters of compounds Fc-Ins9, Fc/FA-Ins6, Fc/FA-Ins7, and Fc/FA-Ins9 from i.v. PK study in rats.

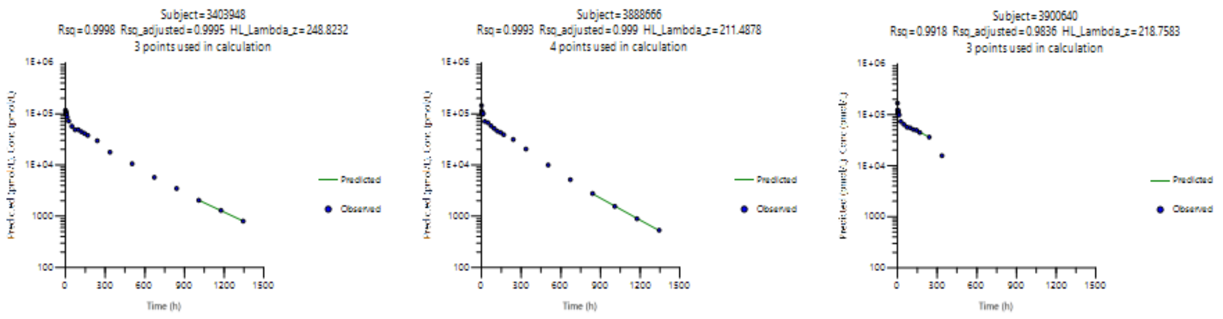


**Figure S4**. Pharmacokinetic profiles from individual animals in dog PK study, Fc/FA-Ins1

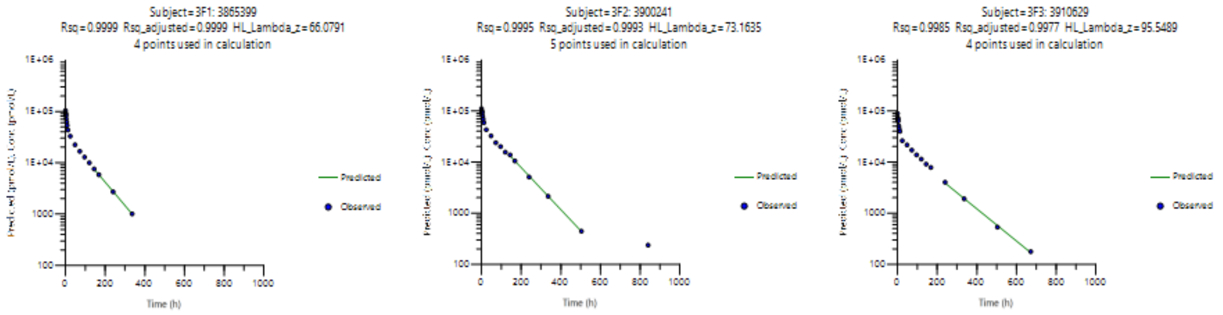


**Figure S5**. Pharmacokinetic profiles from individual animals in dog PK study, Fc-Ins1

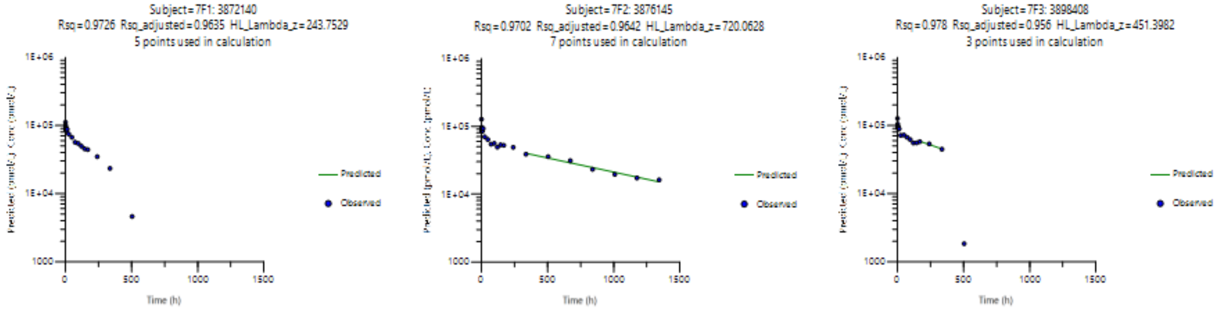


**Figure S6**. Pharmacokinetic profiles from individual animals in dog PK study, Fc/FA-Ins9

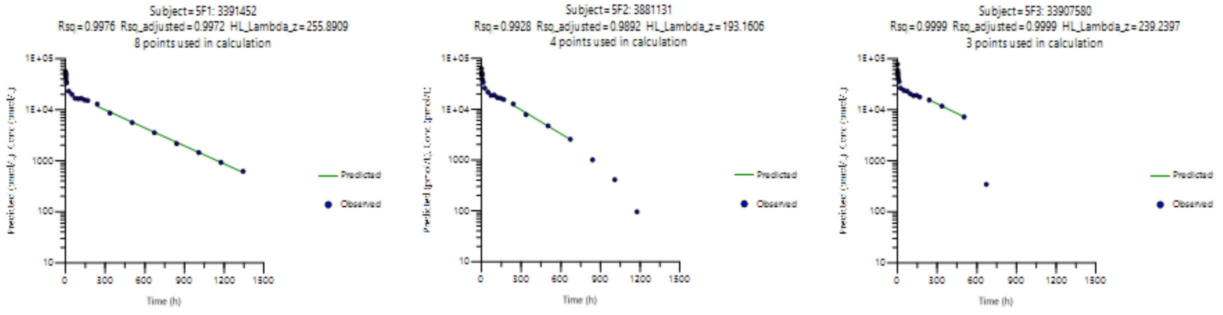


**Figure S7**. Pharmacokinetic profiles from individual animals in dog PK study, Fc-Ins9

**Table S3**. Pharmacokinetic parameters from individual animals in dog PK study

| Analyte | Subject | Dose (pmol) | Rsq | No_points_lambda_z | HL_Lambda_z (h) | AUC_%Extrap_pred (%) | Tmax (h) | Cmax (pmol/L) | C0 (pmol/L) | AUCINF_pred (h*pmol/L) | Vz_pred (L/kg) | Cl_pred (L/h/kg) | Vss_pred (L/kg) |
|---|---|---|---|---|---|---|---|---|---|---|---|---|---|
| Fc/FA-Ins1 | 3400000 | 5000 | 1 | 3 | 249 | 1.47 | 1 | 111000 | 117000 | 19700000 | 0.0912 | 0.000254 | 0.0709 |
| | 3890000 | 5000 | 0.999 | 4 | 211 | 0.787 | 1 | 114000 | 145000 | 20400000 | 0.0749 | 0.000246 | 0.0612 |
| | 3900000 | 5000 | 0.992 | 3 | 219 | 34.7 | 3 | 123000 | 168000 | 24300000 | 0.065 | 0.000206 | 0.0636 |
| | Mean | 5000 | 0.997 | 3.33 | 226 | 12.3 | 1.67 | 116000 | 143000 | 21400000 | 0.077 | 0.000235 | 0.0652 |
| | SD | 0 | 0.00449 | 0.577 | 19.8 | 19.4 | 1.15 | 6240 | 25400 | 2470000 | 0.0132 | 2.56E-05 | 0.00508 |
| | CV% | 0 | 0.45 | 17.3 | 8.74 | 157 | 69.3 | 5.38 | 17.7 | 11.5 | 17.1 | 10.9 | 7.79 |
| | Median | 5000 | 0.999 | 3 | 219 | 1.47 | 1 | 114000 | 145000 | 20400000 | 0.0749 | 0.000246 | 0.0636 |
| | Geometric Mean | 5000 | 0.997 | 3.3 | 226 | 3.42 | 1.44 | 116000 | 142000 | 21300000 | 0.0763 | 0.000234 | 0.0651 |
| | Geometric SD | 1 | 1 | 1.18 | 1.09 | 7.61 | 1.89 | 1.05 | 1.2 | 1.12 | 1.19 | 1.12 | 1.08 |
| | Geometric CV% | 0 | 0.451 | 16.7 | 8.59 | 777 | 70.4 | 5.33 | 18.2 | 11.3 | 17.1 | 11.3 | 7.68 |
| Fc-Ins1 | 3F1: 3865399 | 5000 | 1 | 4 | 66.1 | 2.43 | 1 | 92300 | 104000 | 3950000 | 0.121 | 0.00127 | 0.103 |
| | 3F2: 3900241 | 5000 | 0.999 | 5 | 73.2 | 0.0324 | 1 | 100000 | 111000 | 5910000 | 0.0892 | 0.000845 | 0.0834 |
| | 3F3: 3910629 | 5000 | 0.998 | 4 | 95.5 | 0.553 | 1 | 73800 | 89000 | 4210000 | 0.164 | 0.00119 | 0.133 |
| | Mean | 5000 | 0.999 | 4.33 | 78.3 | 1 | 1 | 88700 | 101000 | 4690000 | 0.125 | 0.0011 | 0.106 |
| | SD | 0 | 0.000731 | 0.577 | 15.4 | 1.26 | 0 | 13500 | 11400 | 1070000 | 0.0373 | 0.000224 | 0.0248 |
| | CV% | 0 | 0.0732 | 13.3 | 19.7 | 125 | 0 | 15.2 | 11.2 | 22.7 | 30 | 20.3 | 23.4 |
| | Median | 5000 | 0.999 | 4 | 73.2 | 0.553 | 1 | 92300 | 104000 | 4210000 | 0.121 | 0.00119 | 0.103 |
| | Geometric Mean | 5000 | 0.999 | 4.31 | 77.3 | 0.351 | 1 | 88000 | 101000 | 4620000 | 0.121 | 0.00108 | 0.104 |
| | Geometric SD | 1 | 1 | 1.14 | 1.21 | 8.97 | 1 | 1.17 | 1.12 | 1.24 | 1.35 | 1.24 | 1.26 |
| | Geometric CV% | 0 | 0.0732 | 12.9 | 19.2 | 1110 | 0 | 15.8 | 11.5 | 22 | 31 | 22 | 23.6 |
| Fc-Ins9 | 5F1: 3391452 | 5000 | 0.998 | 8 | 256 | 2.51 | 1 | 51900 | 56600 | 8620000 | 0.214 | 0.00058 | 0.203 |
| | 5F2: 3881131 | 5000 | 0.993 | 4 | 193 | 1.48 | 1 | 52900 | 63200 | 7830000 | 0.178 | 0.000639 | 0.166 |
| | 5F3: 33907580 | 5000 | 1 | 3 | 239 | 15 | 1 | 59800 | 77900 | 10200000 | 0.169 | 0.000488 | 0.163 |
| | Mean | 5000 | 0.997 | 5 | 229 | 6.32 | 1 | 54900 | 65900 | 8900000 | 0.187 | 0.000569 | 0.177 |
| | SD | 0 | 0.00365 | 2.65 | 32.5 | 7.51 | 0 | 4300 | 10900 | 1230000 | 0.024 | 7.58E-05 | 0.0224 |
| | CV% | 0 | 0.366 | 52.9 | 14.2 | 119 | 0 | 7.84 | 16.5 | 13.8 | 12.9 | 13.3 | 12.6 |
| | Median | 5000 | 0.998 | 4 | 239 | 2.51 | 1 | 52900 | 63200 | 8620000 | 0.178 | 0.00058 | 0.166 |
| | Geometric Mean | 5000 | 0.997 | 4.58 | 228 | 3.82 | 1 | 54800 | 65300 | 8840000 | 0.186 | 0.000565 | 0.176 |
| | Geometric SD | 1 | 1 | 1.66 | 1.16 | 3.36 | 1 | 1.08 | 1.18 | 1.15 | 1.13 | 1.15 | 1.13 |
| | Geometric CV% | 0 | 0.367 | 53.8 | 14.8 | 183 | 0 | 7.7 | 16.3 | 13.7 | 12.6 | 13.7 | 12.3 |
| Fc/FA-Ins9 | 7F1: 3872140 | 5000 | 0.973 | 5 | 244 | 24.6 | 1 | 99300 | 111000 | 23900000 | 0.0736 | 0.000209 | 0.0698 |
| | 7F2: 3876145 | 5000 | 0.97 | 7 | 720 | 26.4 | 1 | 96700 | 127000 | 59700000 | 0.0871 | 8.38E-05 | 0.0838 |
| | 7F3: 3898408 | 5000 | 0.978 | 3 | 451 | 48.9 | 1 | 105000 | 127000 | 46500000 | 0.07 | 0.000107 | 0.0707 |
| | Mean | 5000 | 0.974 | 5 | 472 | 33.3 | 1 | 100000 | 122000 | 43400000 | 0.0769 | 0.000133 | 0.0748 |
| | SD | 0 | 0.00398 | 2 | 239 | 13.5 | 0 | 4250 | 8870 | 18100000 | 0.00901 | 6.66E-05 | 0.00782 |
| | CV% | 0 | 0.409 | 40 | 50.6 | 40.7 | 0 | 4.23 | 7.3 | 41.7 | 11.7 | 49.9 | 10.5 |
| | Median | 5000 | 0.973 | 5 | 451 | 26.4 | 1 | 99300 | 127000 | 46500000 | 0.0736 | 0.000107 | 0.0707 |
| | Geometric Mean | 5000 | 0.974 | 4.72 | 429 | 31.7 | 1 | 100000 | 121000 | 40500000 | 0.0765 | 0.000124 | 0.0745 |
| | Geometric SD | 1 | 1 | 1.53 | 1.72 | 1.46 | 1 | 1.04 | 1.08 | 1.6 | 1.12 | 1.6 | 1.11 |
| | Geometric CV% | 0 | 0.409 | 44.7 | 58.6 | 39.2 | 0 | 4.21 | 7.48 | 50.1 | 11.5 | 50.1 | 10.2 |

**In-vitro insulin receptor activity assays**

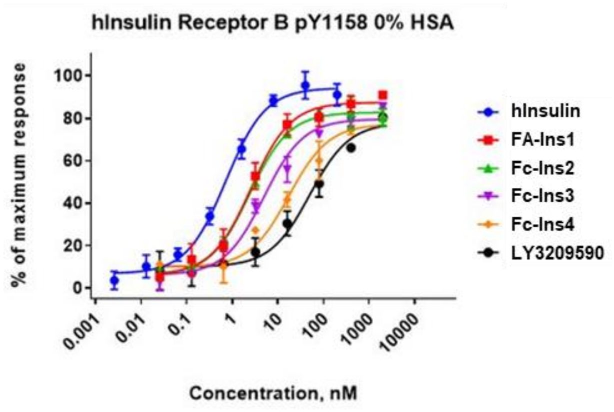


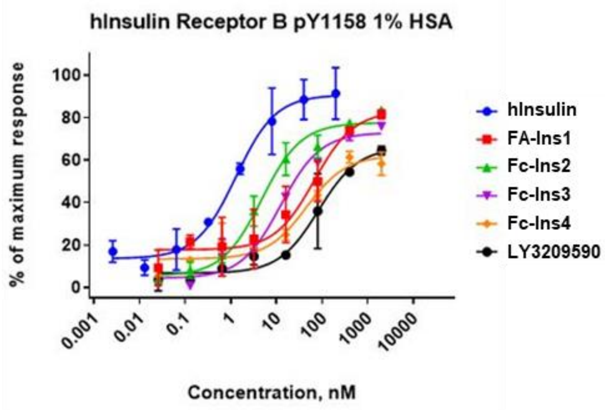


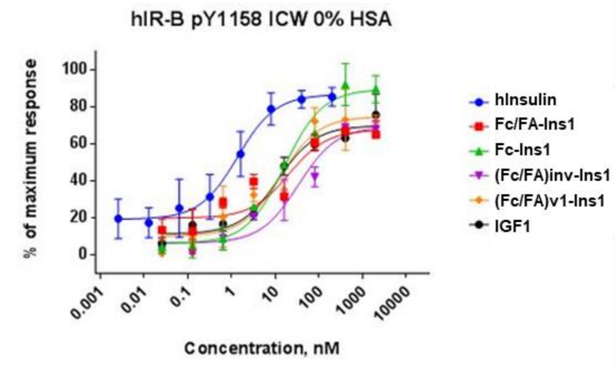


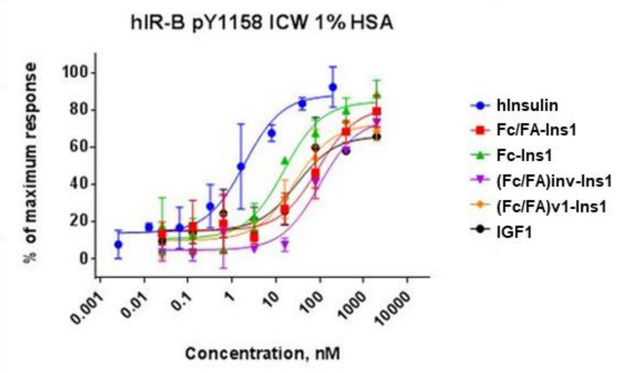


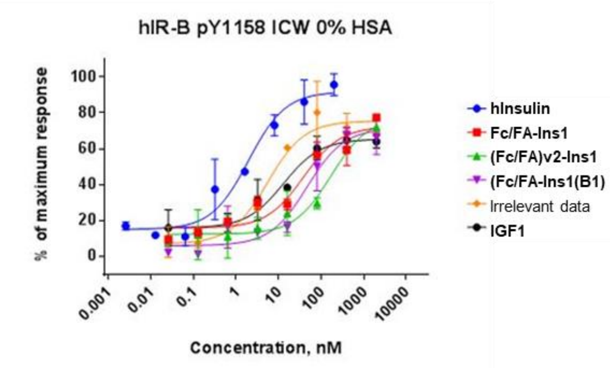


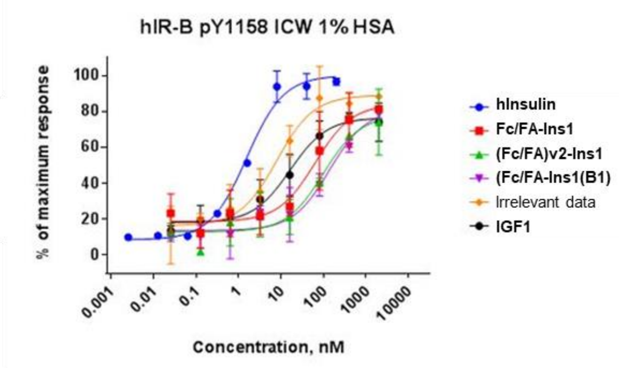


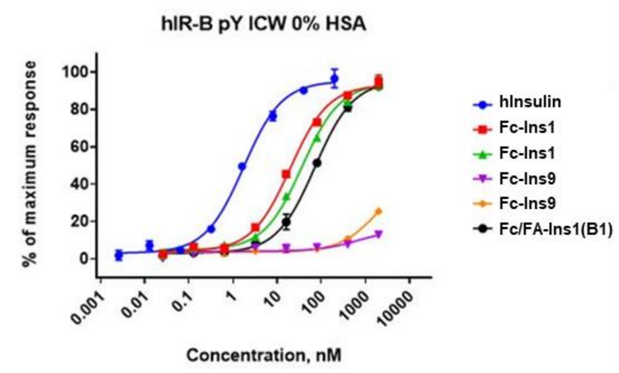


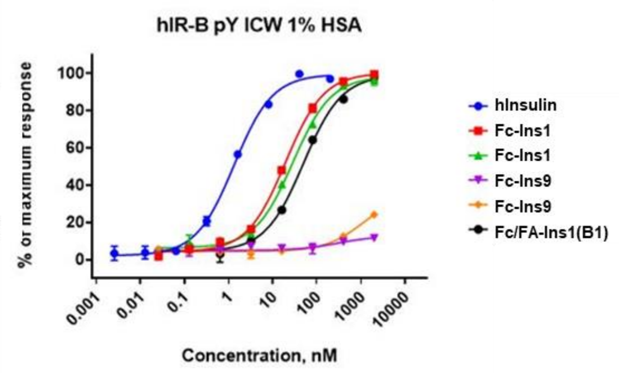

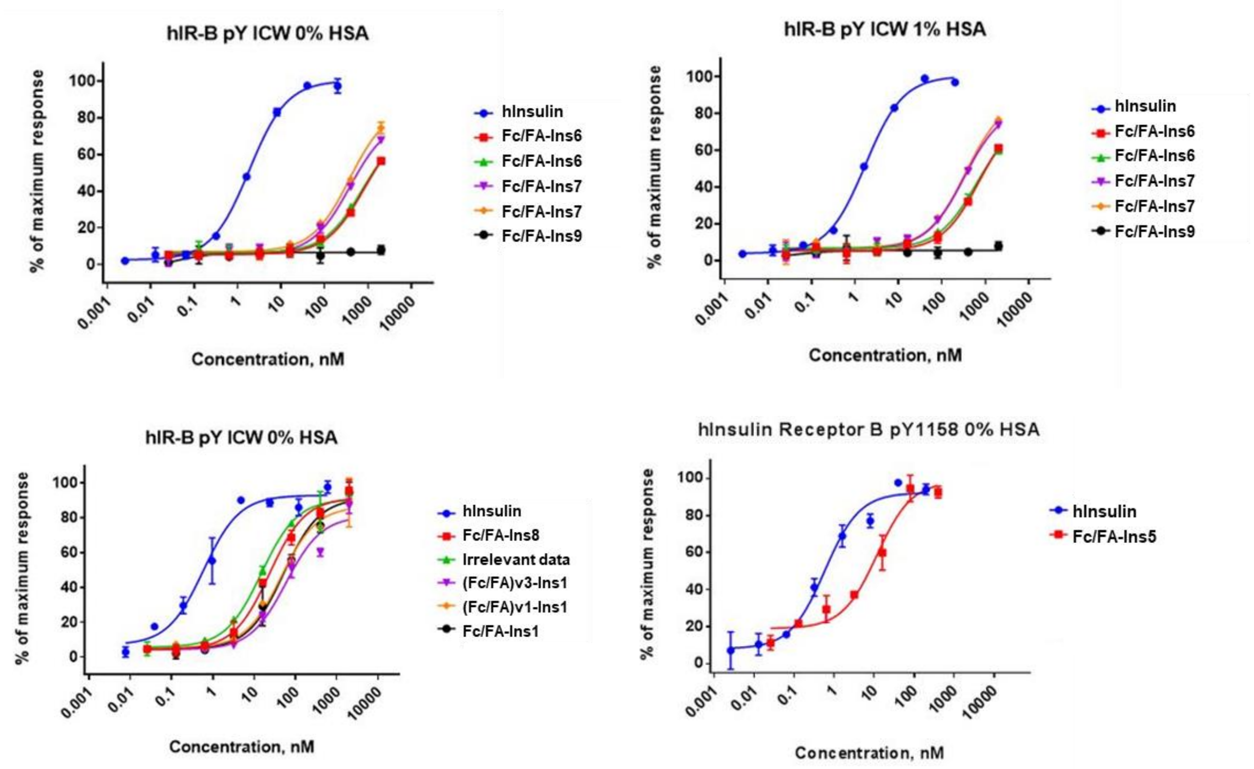


**Figure S8**. Representative dose-response curves from in-vitro insulin receptor activity assays. Insulin receptor phosphorylation was measured in an indirect ELISA assay using HEK293 cells overexpressing B-isoform of human insulin receptor (hIR-B). The EC50 values and potencies relative to native insulin used in the same assay are reported in the Figure 1.

## Representative synthesis

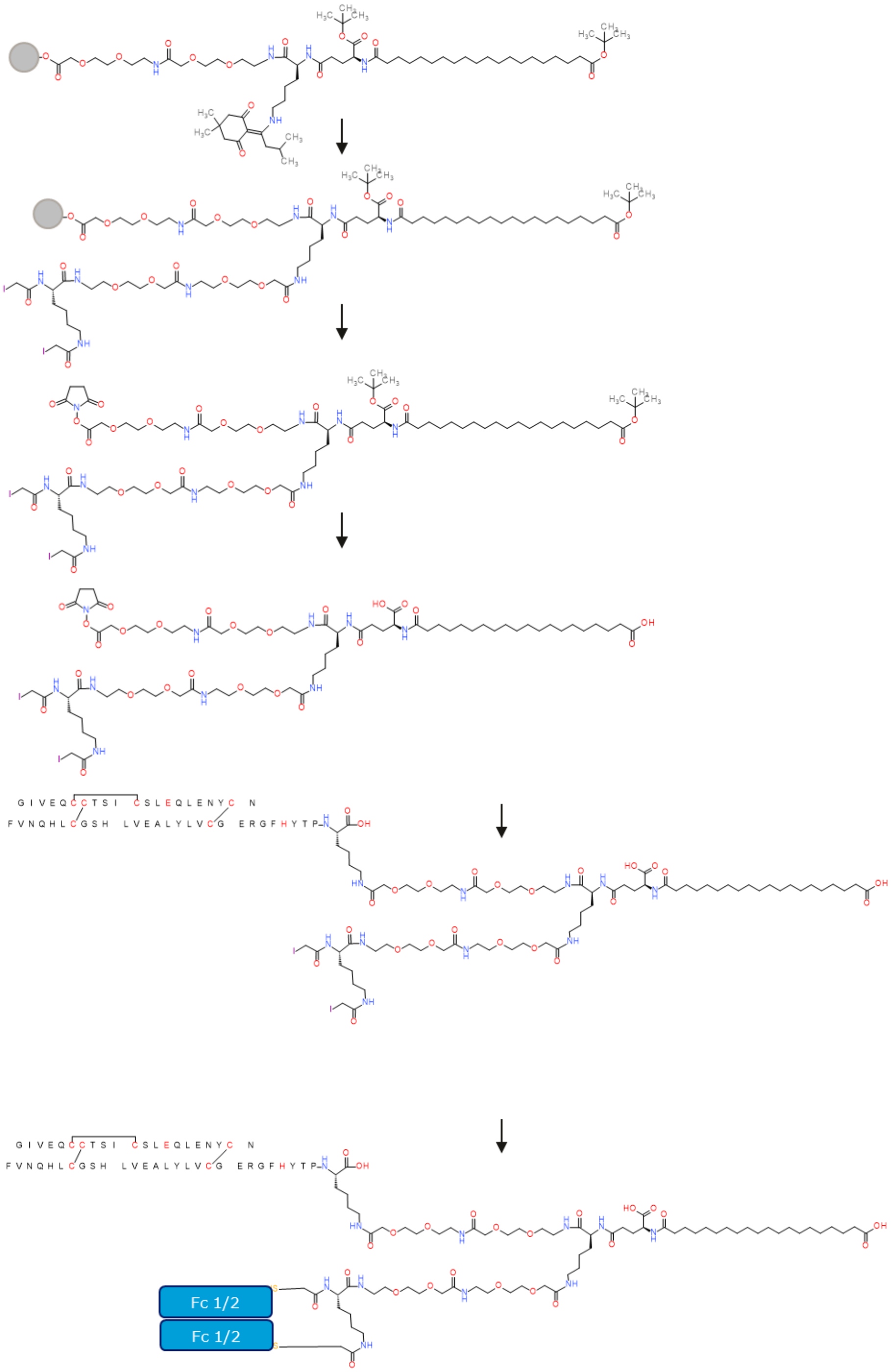

**Figure S9.** A generalized synthetic scheme for preparation of Fc/FA-Ins1.

## Structures and characterizations

**Table S4.** LCMS characterization of the insulin compounds prepared in this study.

| Compound | Calc mass | Obs. Mass |
|---|---|---|
| Fc/FA-Ins1 | 58807 | 58807 |
| Fc-Ins1 | 56771 | 56774 |
| Fc/FA-Ins1(B1) | 58807 | 58809 |
| Fc/FA-Ins5 | 58964 | 58962 |
| Fc/FA-Ins6 | 58758 | 58760 |
| Fc/FA-Ins7 | 58782 | 58782 |
| Fc/FA-Ins8 | 58851 | 58851 |
| (Fc/FA)inv-Ins1 | 58801 | 58809 |
| (Fc/FA)v1-Ins1 | 57936 | 57933 |
| (Fc/FA)v2-Ins1 | 57936 | 57939 |
| (Fc/FA)v3-Ins1 | 57645 | 57645 |
| (Fc/FA)half-Ins1 | 34099 | 34101 |
| Fc/FA-Ins9 | 58736 | 58740 |
| Fc-Ins9 | 56701 | 56700 |

Fc/FA-Ins1

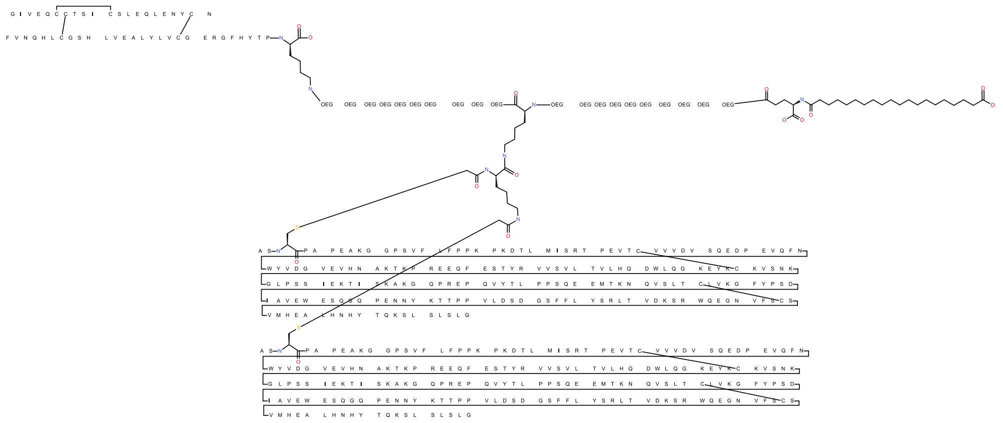

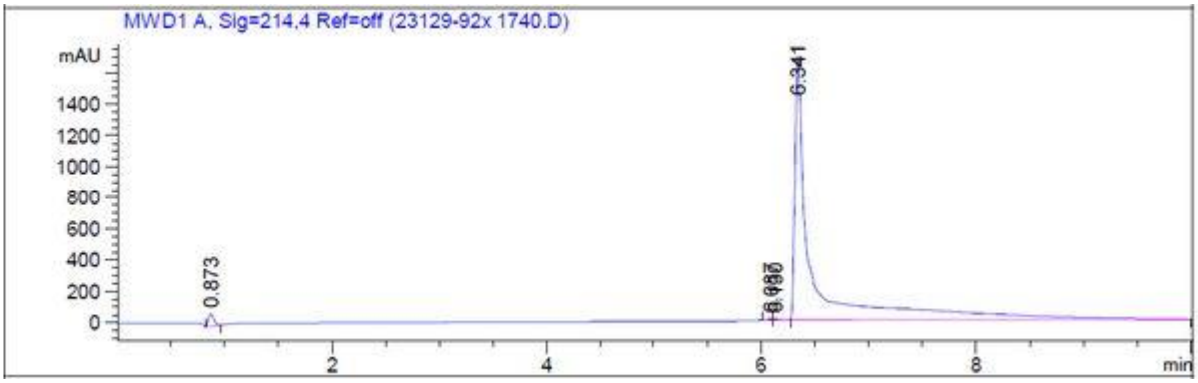


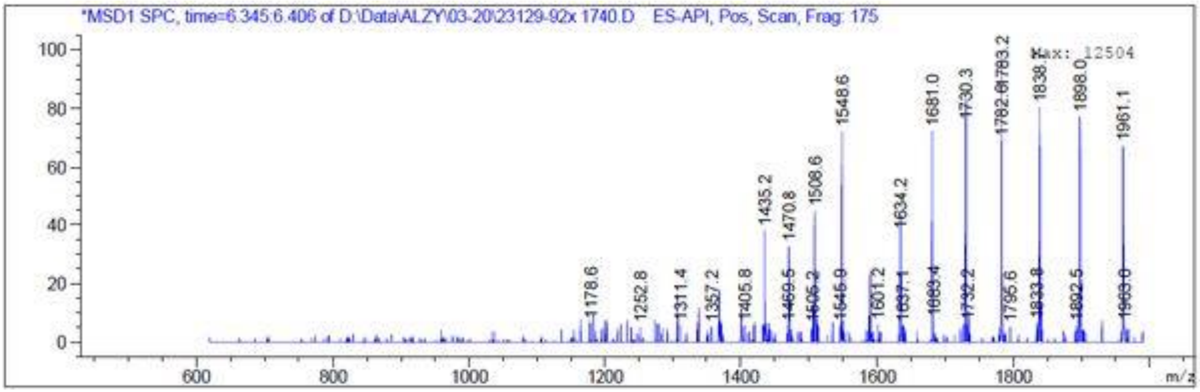


MW exp 58807, obs 58807

Fc-Ins1

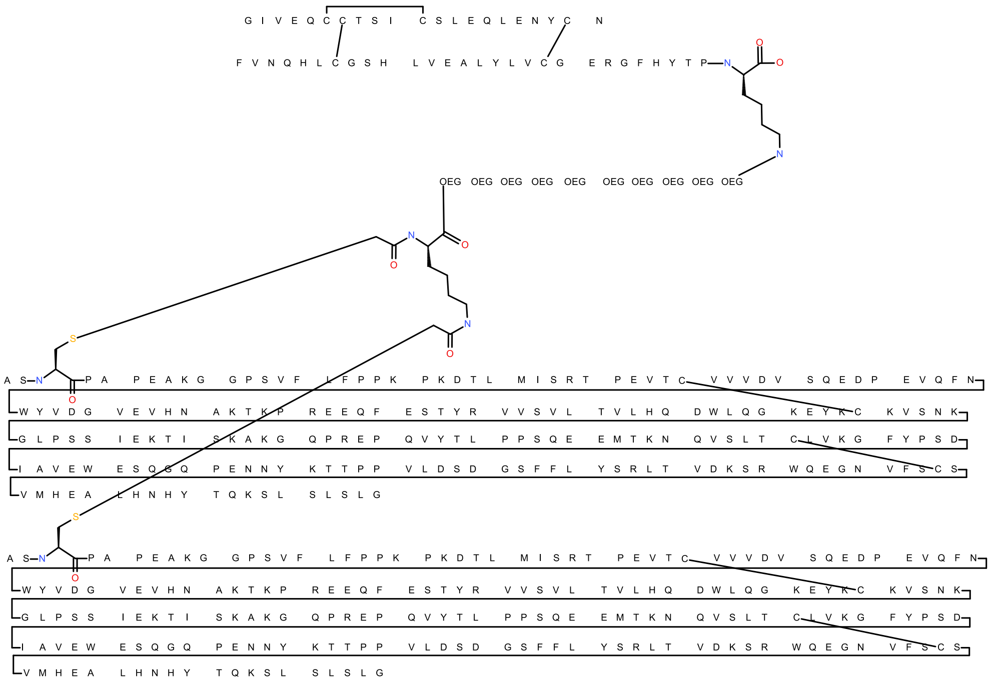
G I V E Q C C T S I C S L E Q L E N Y C N
F V N Q H L C G S H L V E A L Y L V C G E R G F H Y T P
OEG OEG OEG OEG OEG OEG OEG OEG OEG OEG
A S PA PEAKG GPSVF LFPPK PKDTL MISRT PEVTC VVVDV SQEDP EVQFN
WYVDG VEVHN AKTKP REEQF ESTYR VVSVL TVLHQ DWLQG KEYKC KVSNK
GLPSS IEKTI SKAKG QPREP QVYTL PPSQE EMTKN QVSLT CLVKG FYPSD
IAVEW ESQGQ PENNY KTTPP VLDSD GSFFL YSRLT VDKSR WQEGN VFSCS
VMHEA LHNHY TQKSL SLSLG
A S PA PEAKG GPSVF LFPPK PKDTL MISRT PEVTC VVVDV SQEDP EVQFN
WYVDG VEVHN AKTKP REEQF ESTYR VVSVL TVLHQ DWLQG KEYKC KVSNK
GLPSS IEKTI SKAKG QPREP QVYTL PPSQE EMTKN QVSLT CLVKG FYPSD
IAVEW ESQGQ PENNY KTTPP VLDSD GSFFL YSRLT VDKSR WQEGN VFSCS
VMHEA LHNHY TQKSL SLSLG

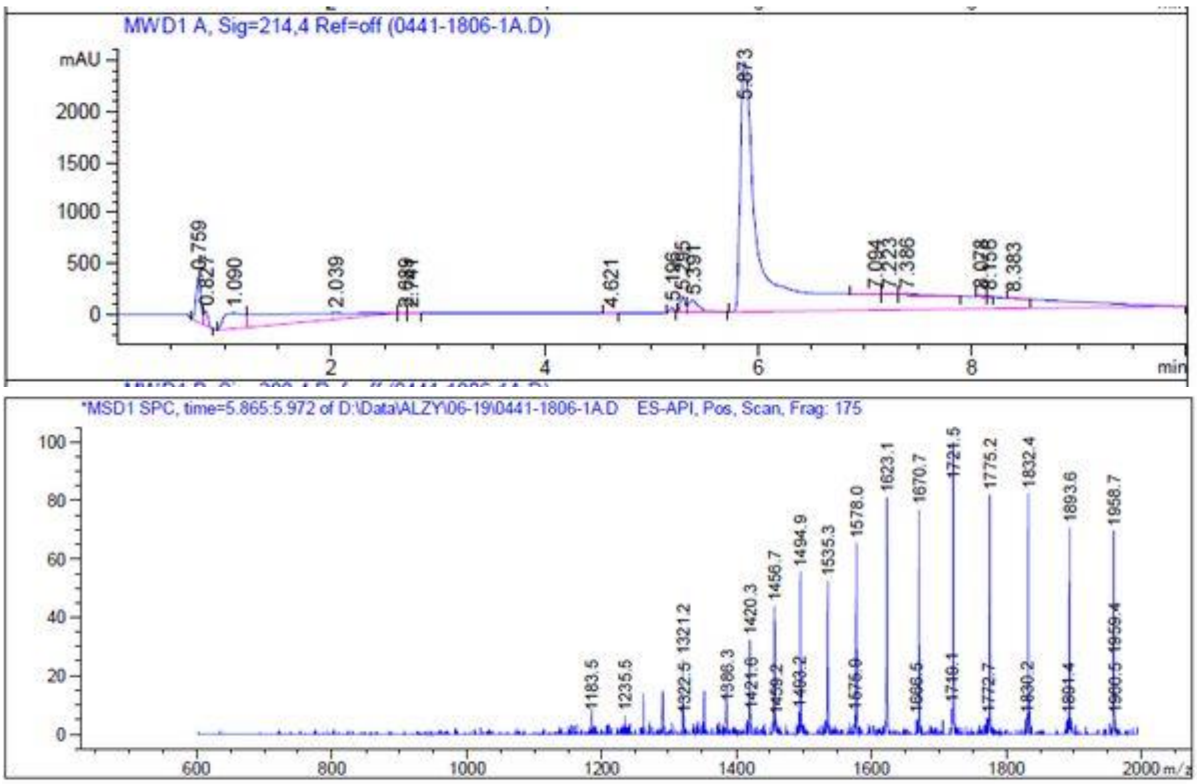
MWD1 A, Sig=214,4 Ref=off (0441-1806-1A.D)
mAU
5.873
0.759
0.827
1.090
2.039
4.621
5.391
7.094
7.223
7.386
8.078
8.156
8.383
min
*MSD1 SPC, time=5.865:5.972 of D:\Data\ALZY\06-19\0441-1806-1A.D ES-API, Pos, Scan, Frag: 175
1183.5
1235.5
1321.2
1322.5
1420.3
1456.7
1494.9
1535.3
1578.0
1623.1
1670.7
1721.5
1775.2
1832.4
1893.6
1958.7
m/z

MW exp 56771, obs 55774

Fc/FA-Ins1(B1)

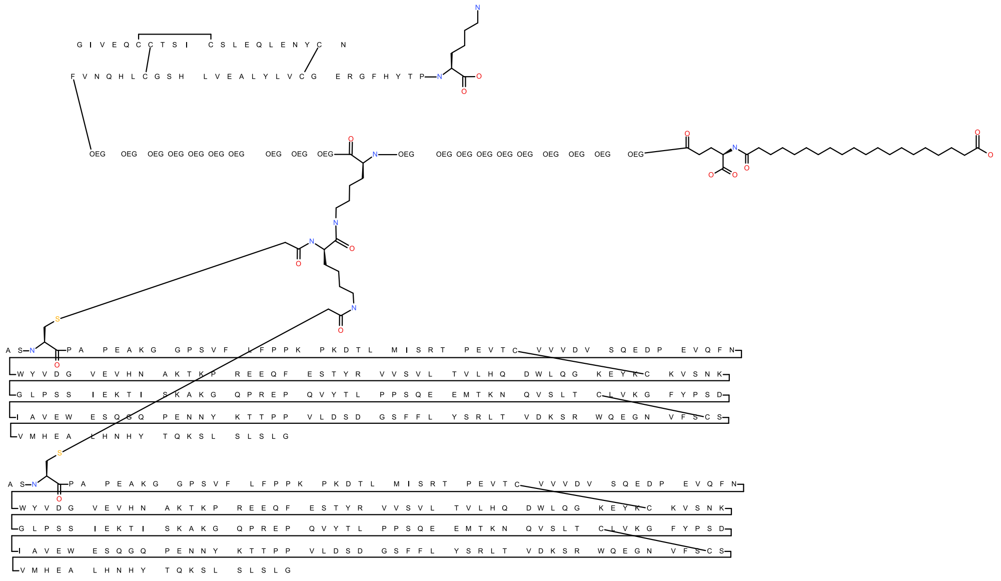

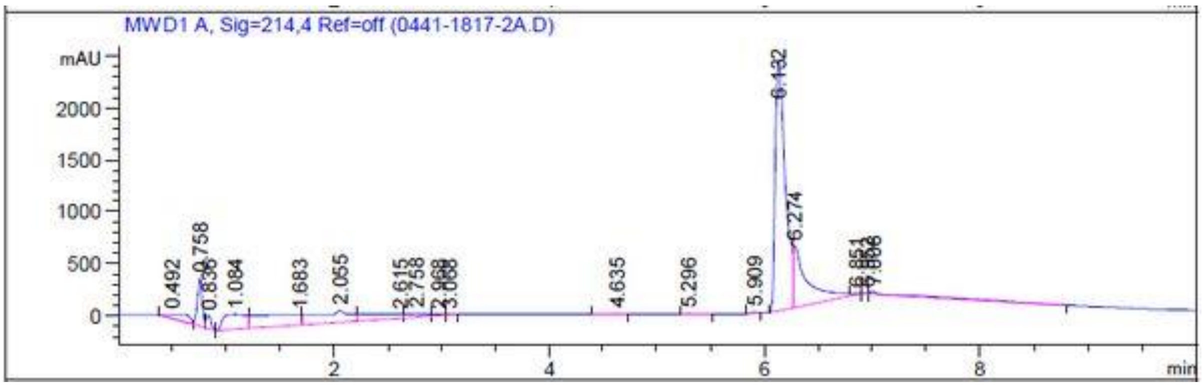


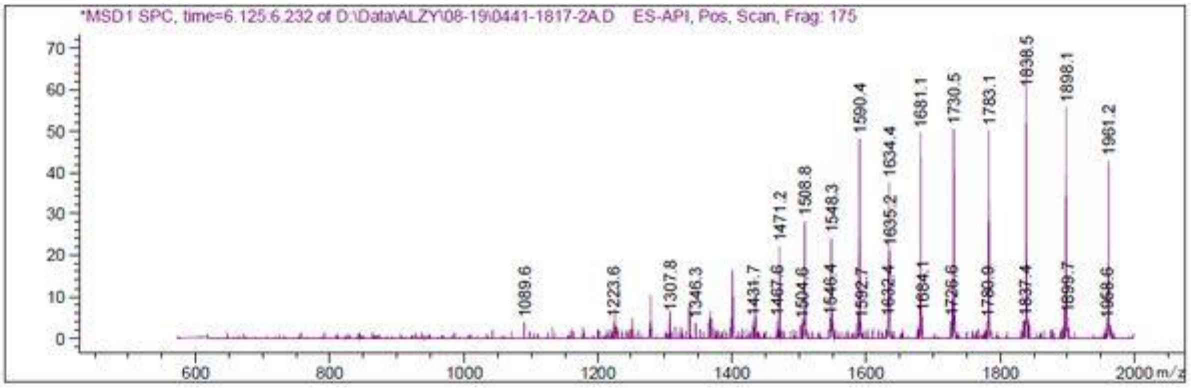


MW exp 58807, obs 58809

Fc/FA-Ins5

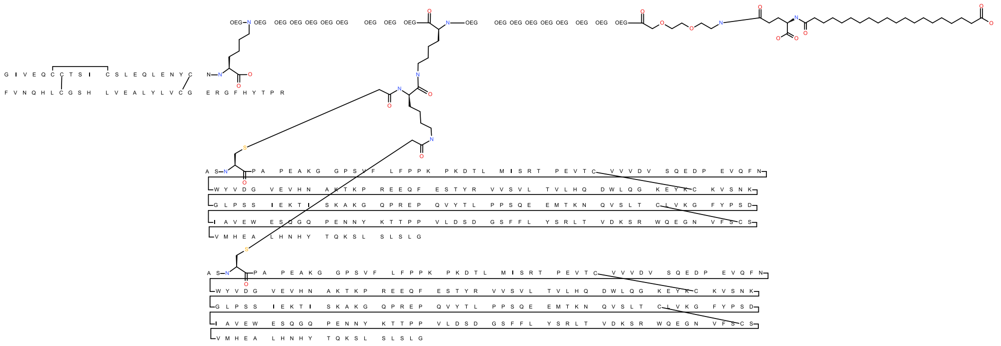

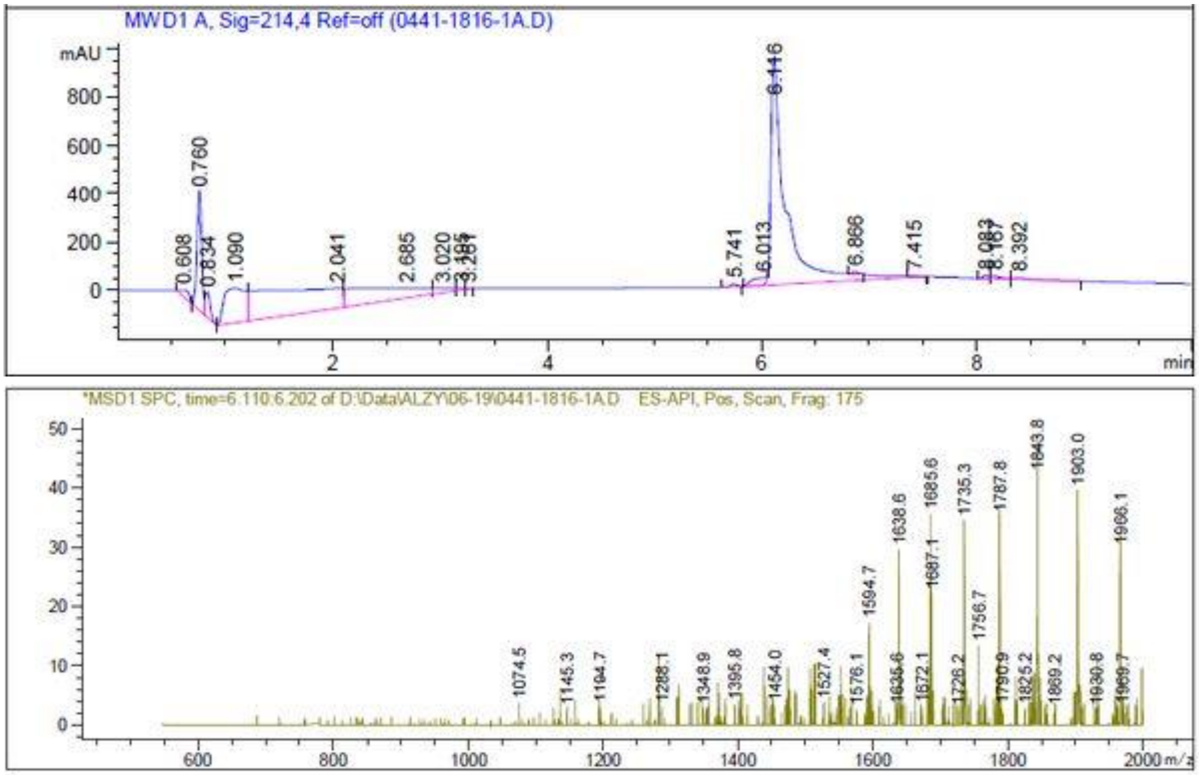


Exp 58964, obs 58962

Fc/FA-Ins6

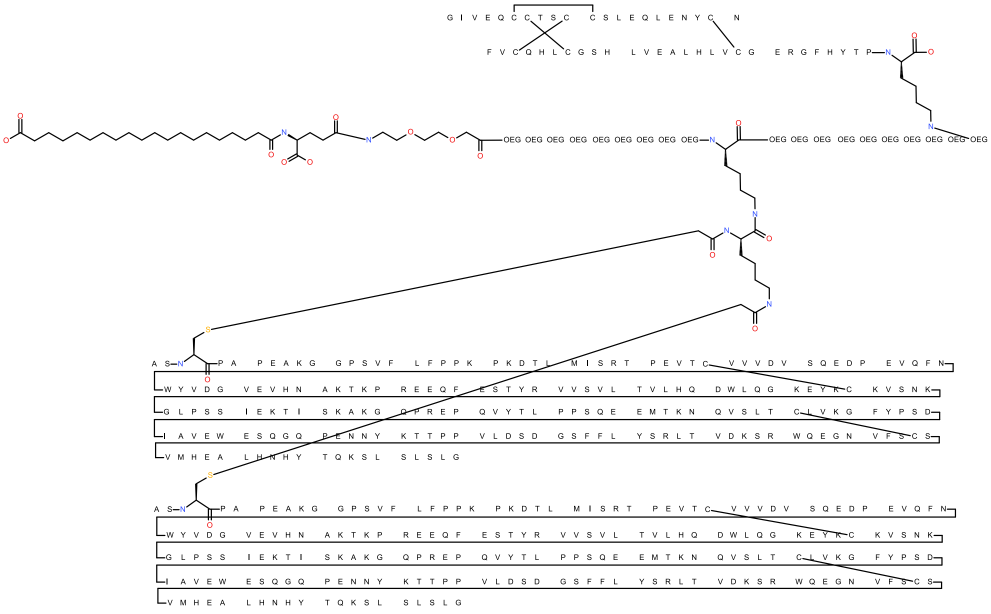

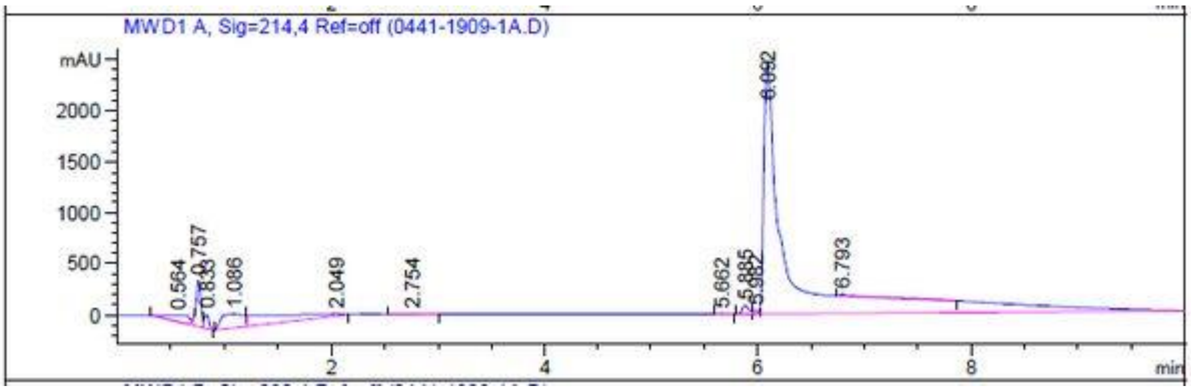


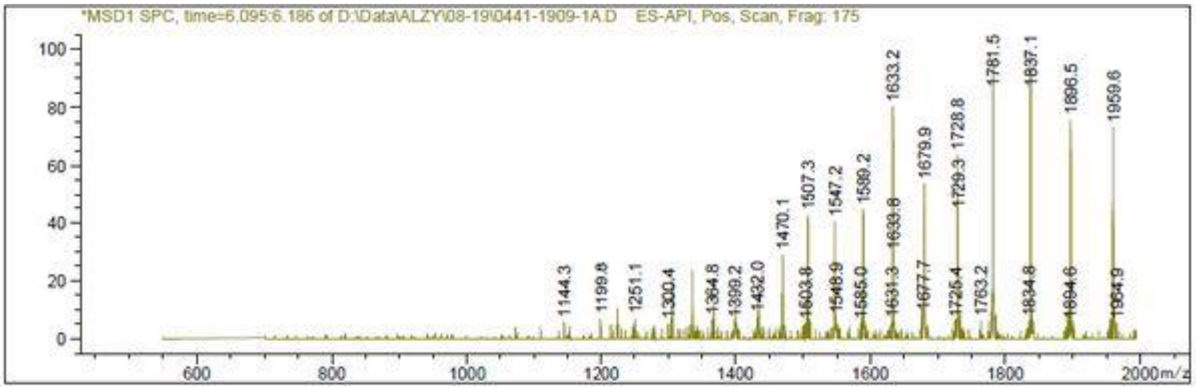


MW exp 58758, obs 58760

Fc/FA-Ins7

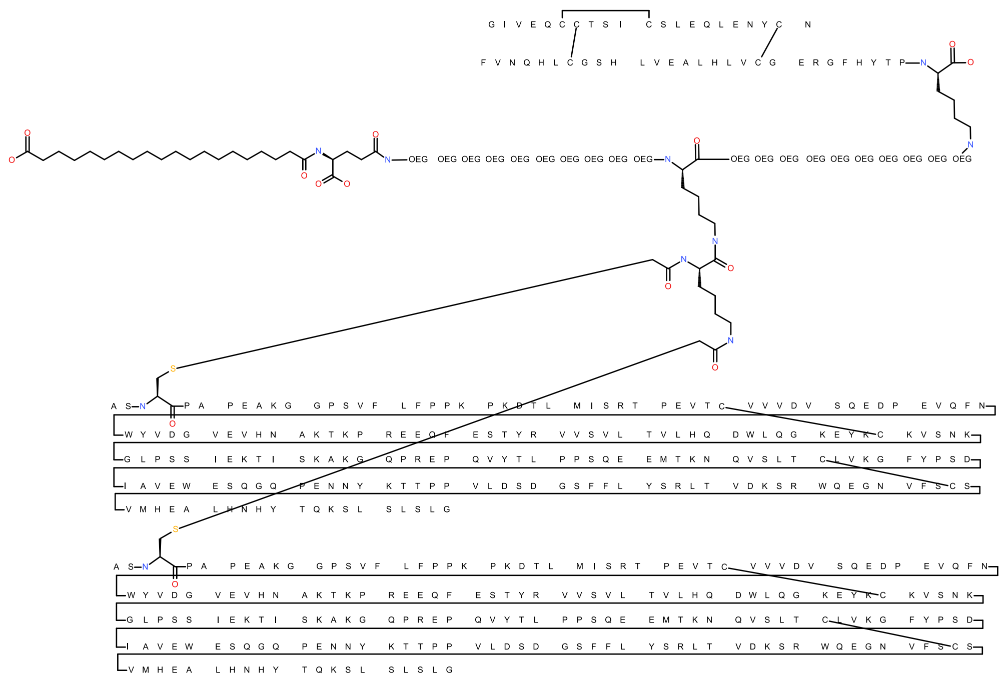

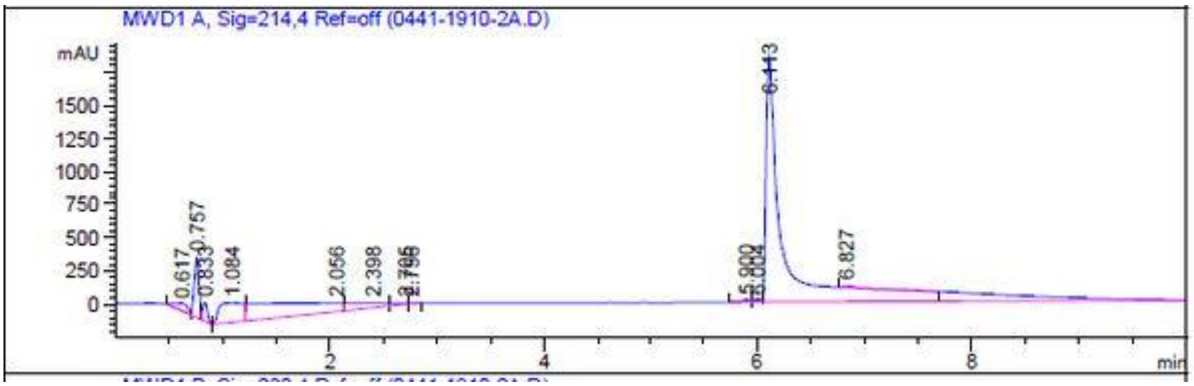


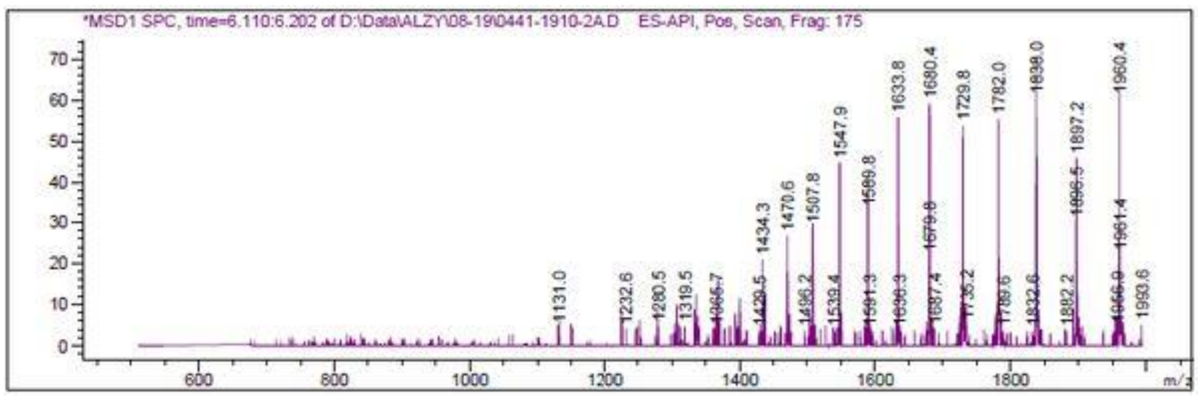


MW exp 58782, obs 58782

Fc/FA-Ins8

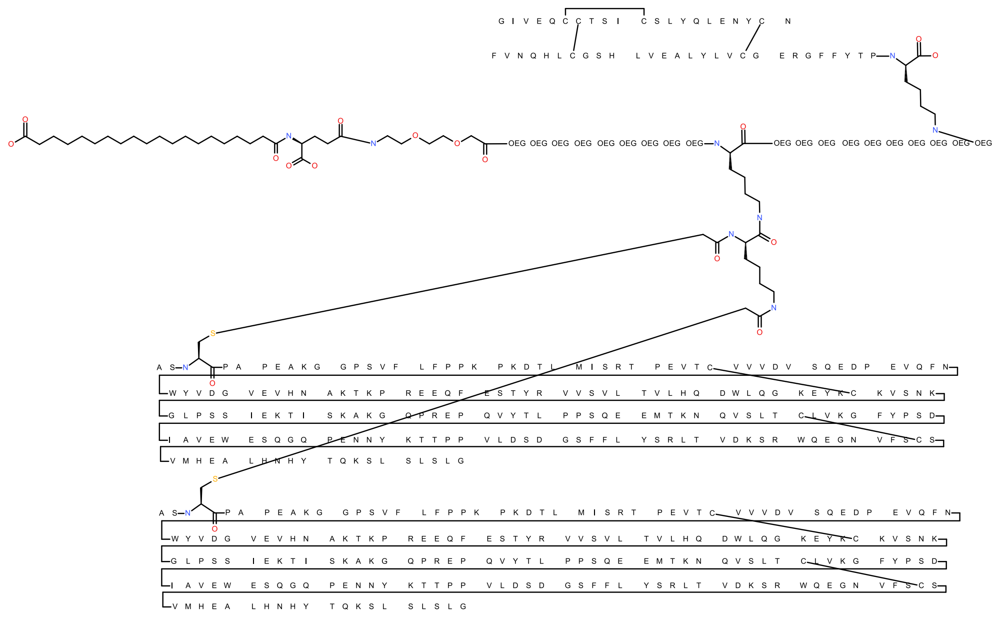

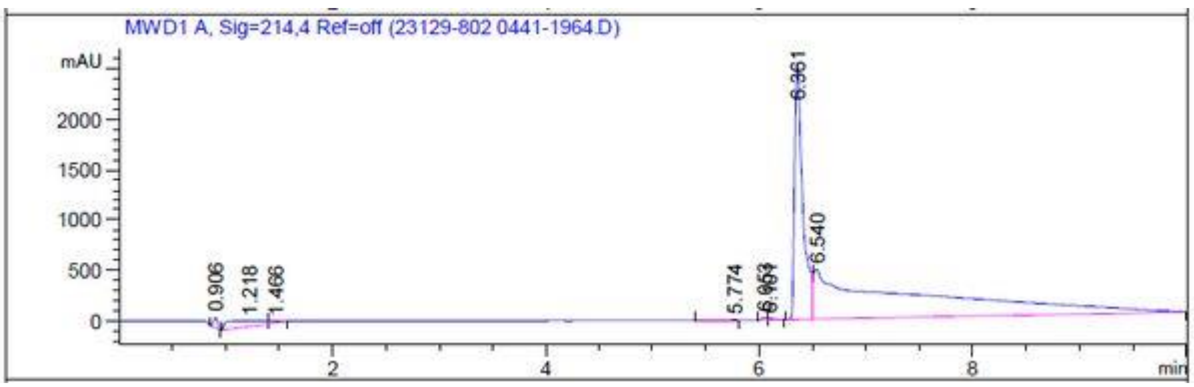


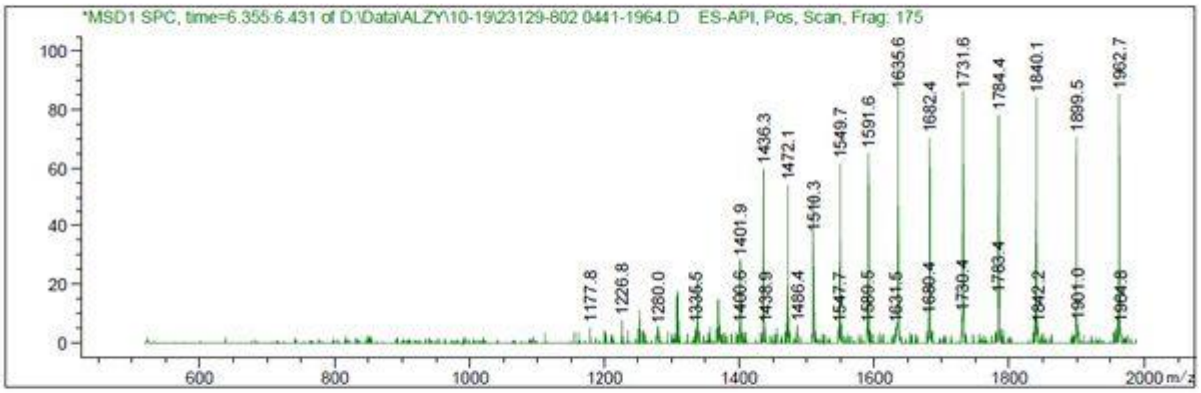


MW exp 58851, MW obs 58851

(Fc/FA)inv-Ins1

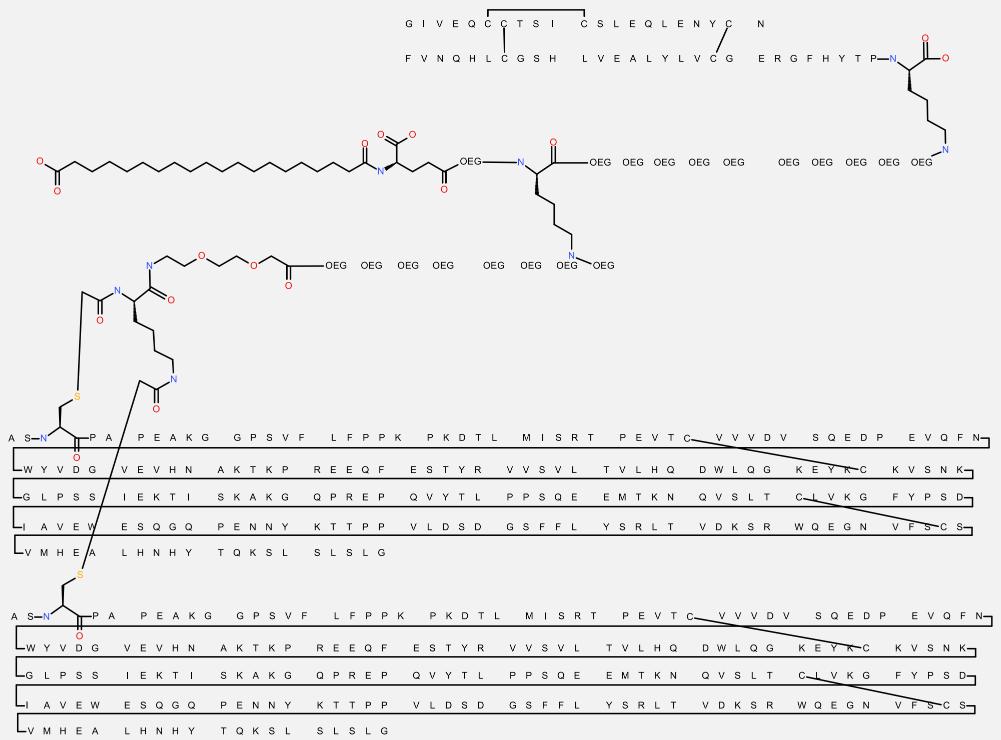


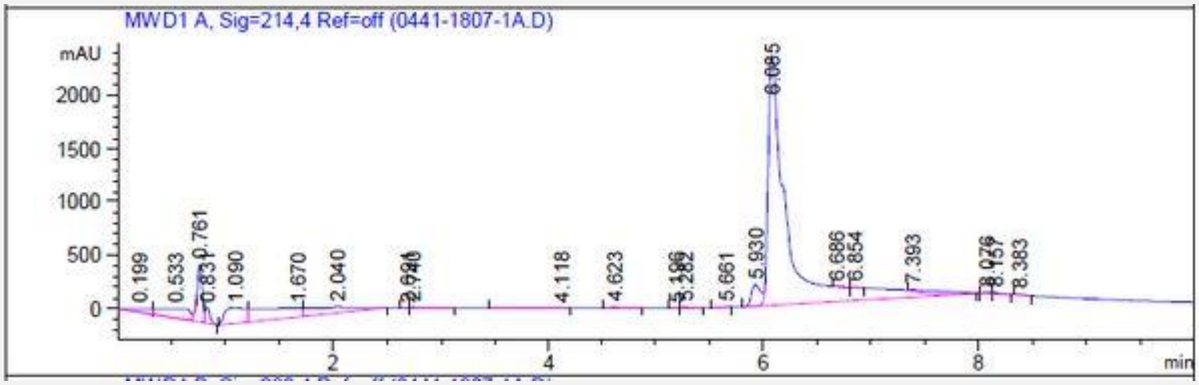

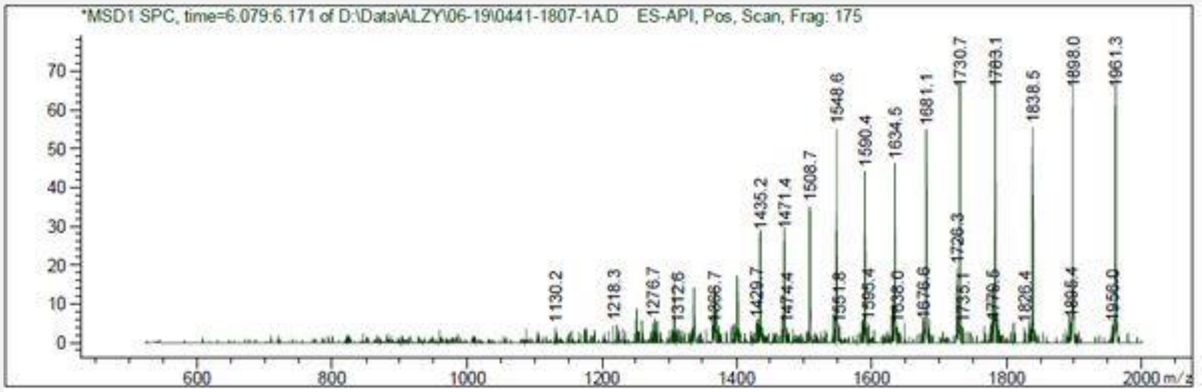


MW exp 58807, obs 58809

(Fc/FA)v1-Ins1

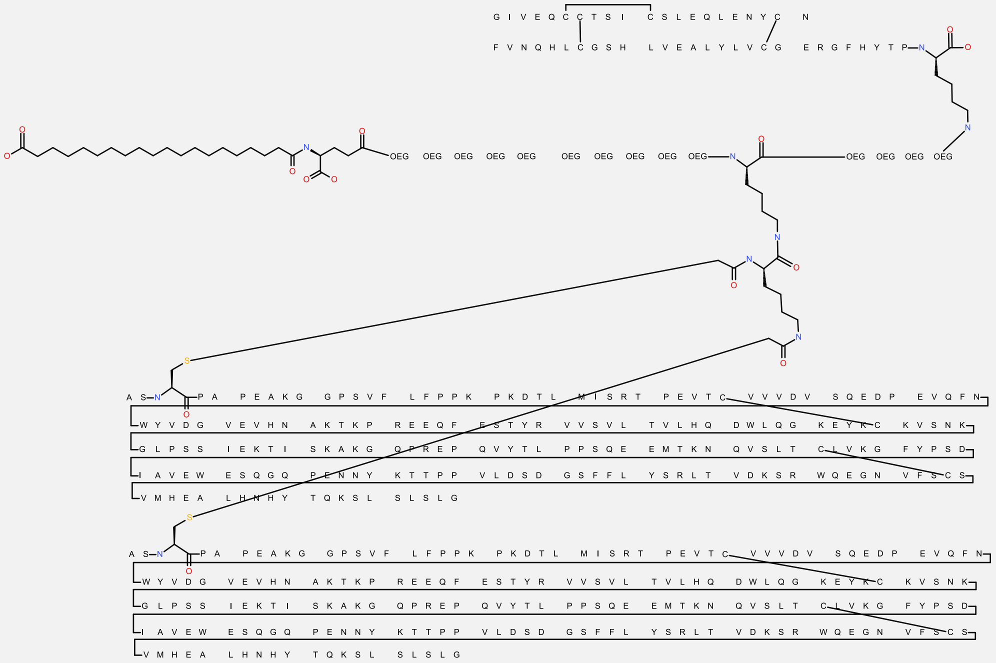

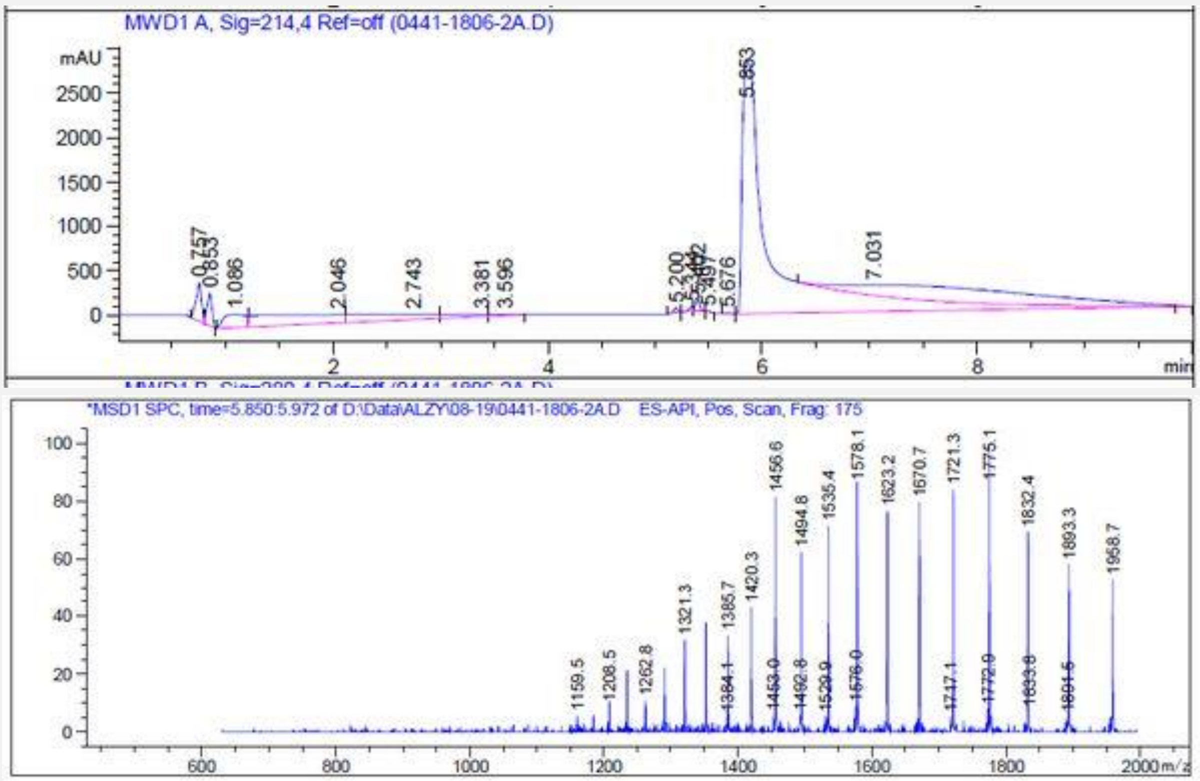


MW exp 57936, obs 57933

(Fc/FA)v2-Ins1

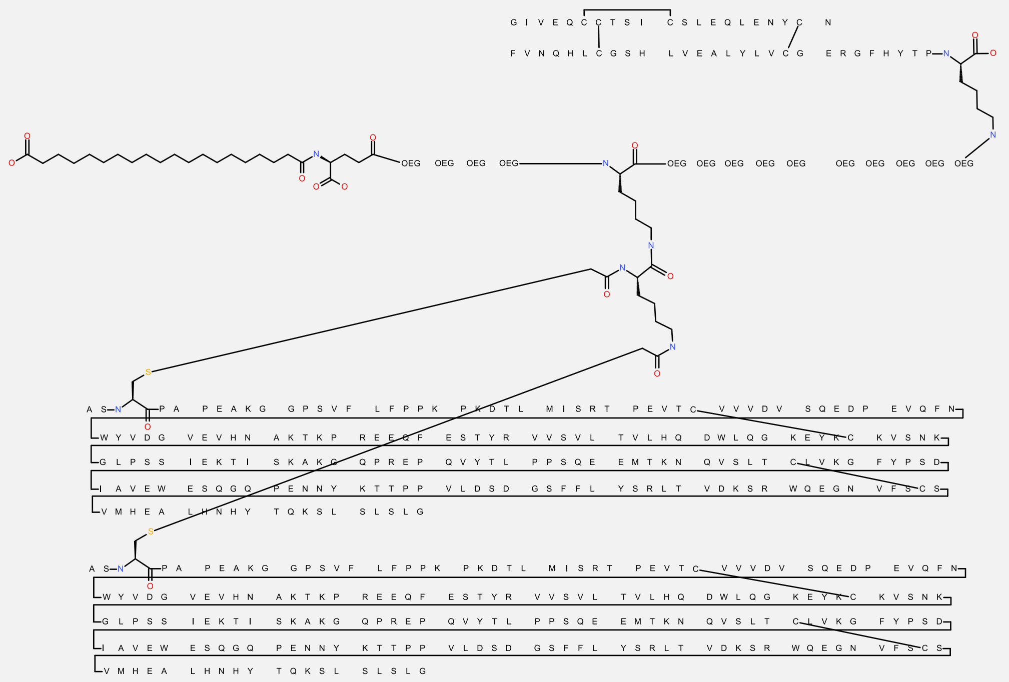

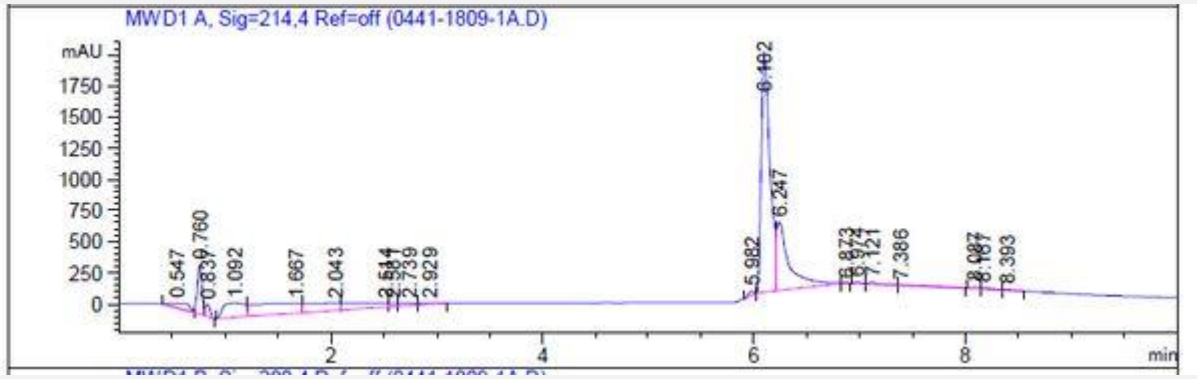


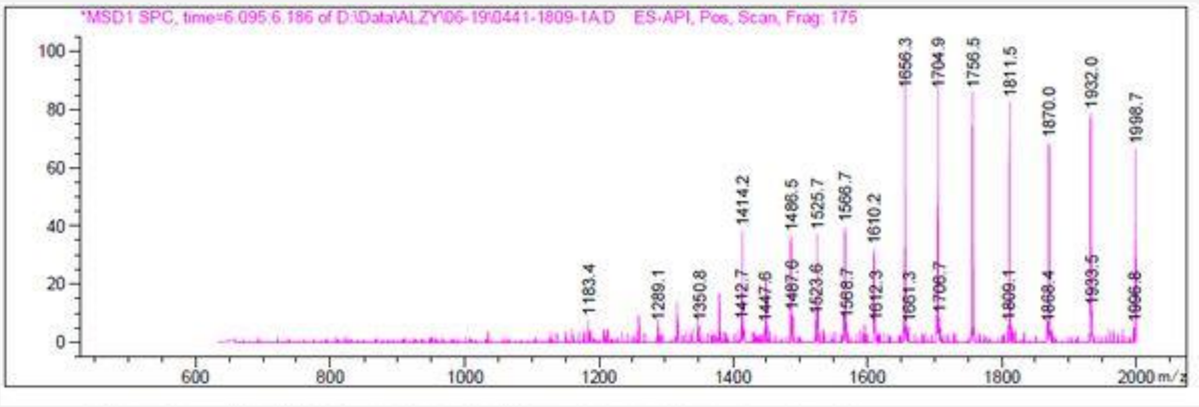


MW 57936, obs 57939

(Fc/FA)v3-Ins1

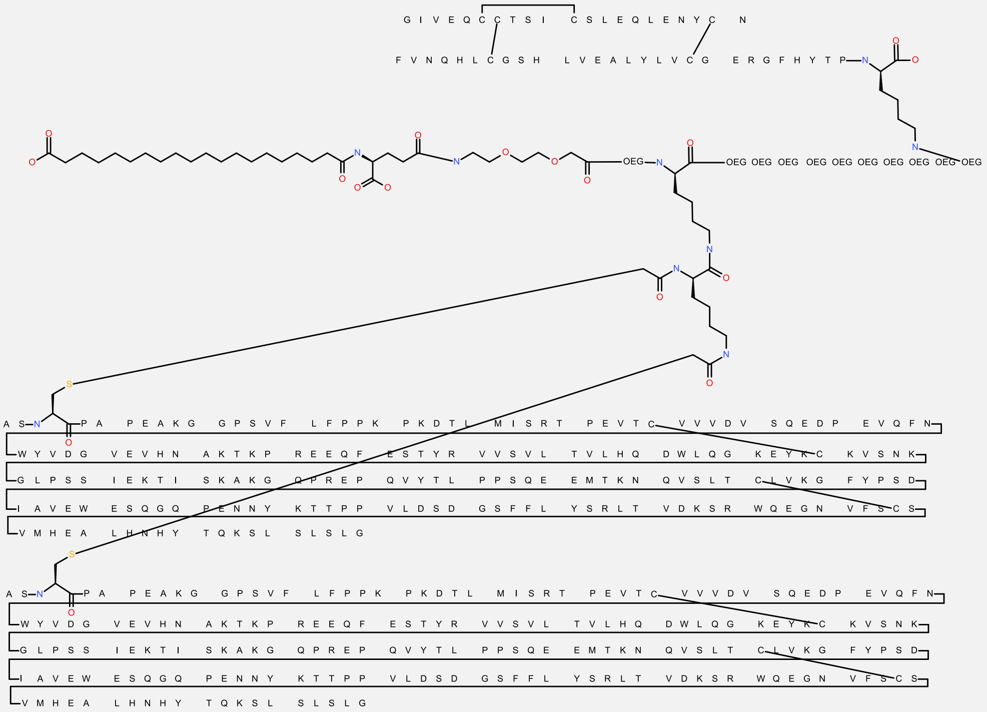

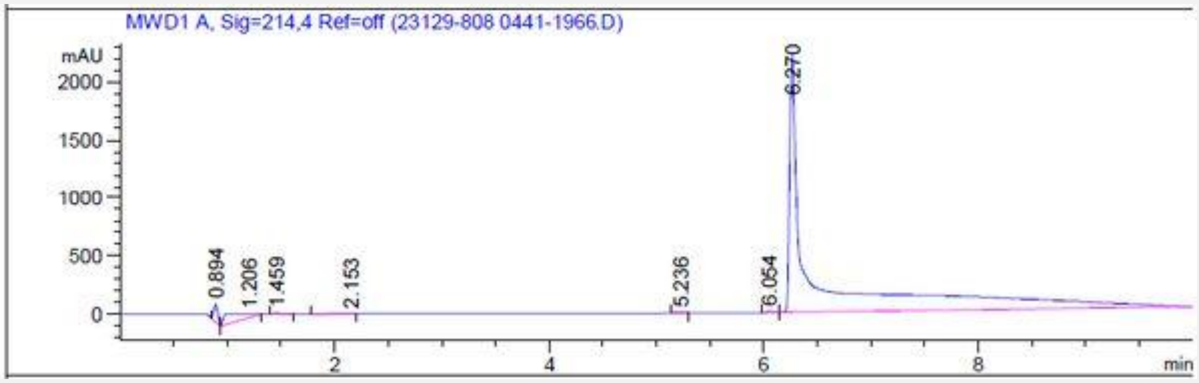
MWD1 A, Sig=214,4 Ref=off (23129-808 0441-1966.D)
mAU
2000
1500
1000
500
0
0.894
1.206
1.459
2.153
5.236
6.054
6.270
2
4
6
8
min

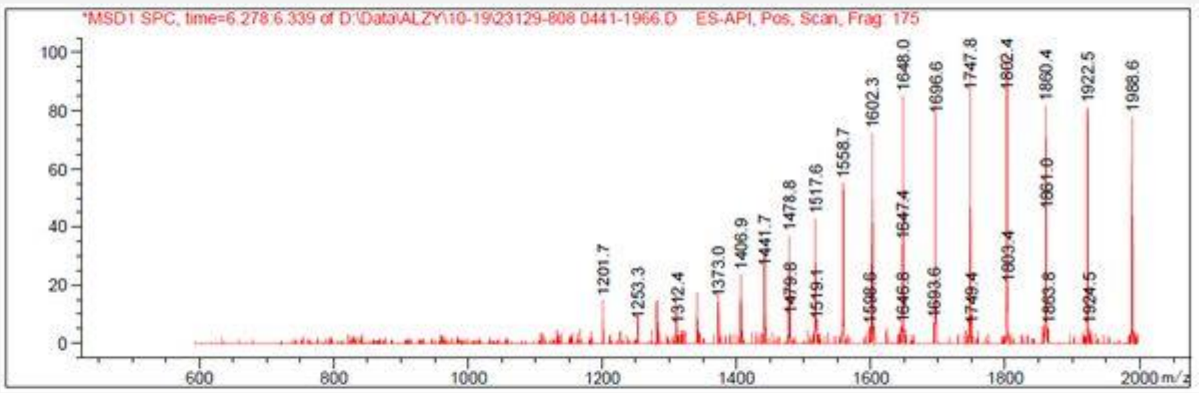


MW exp 57645, obs 57645

(Fc/FA)half-Ins1

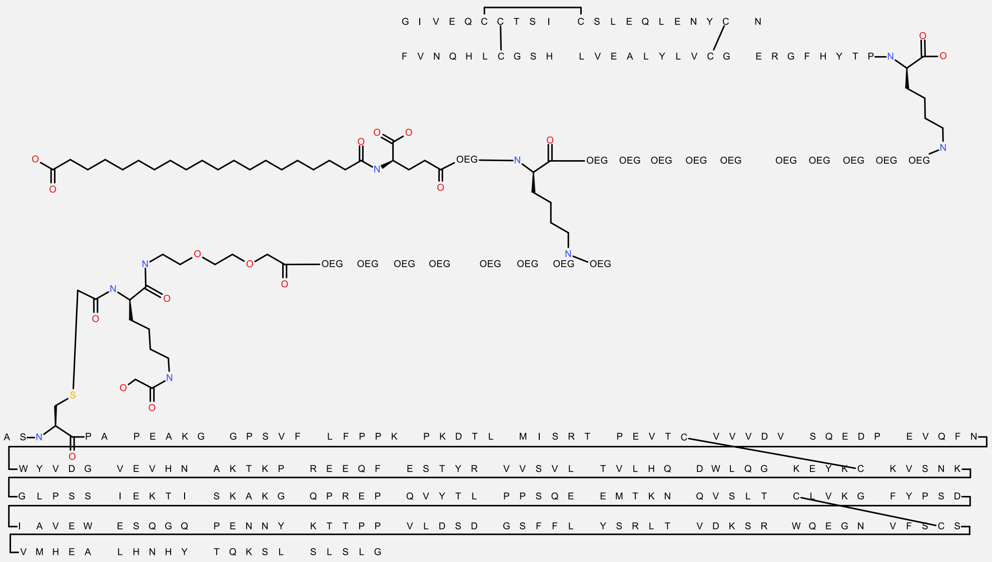

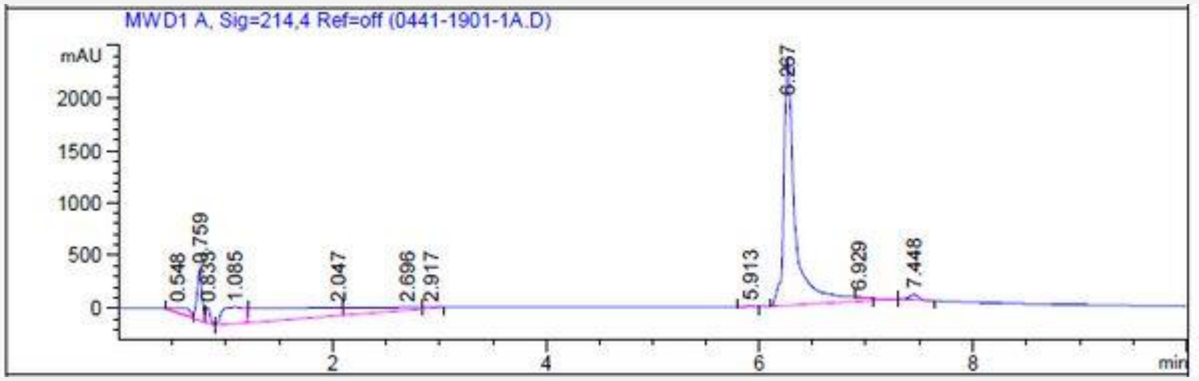

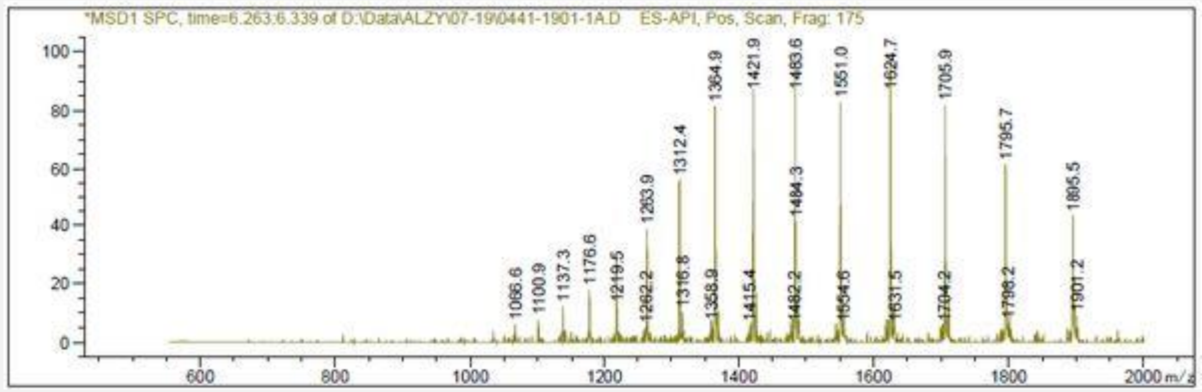


MW 34099, obs 34101

Fc/FA-Ins9

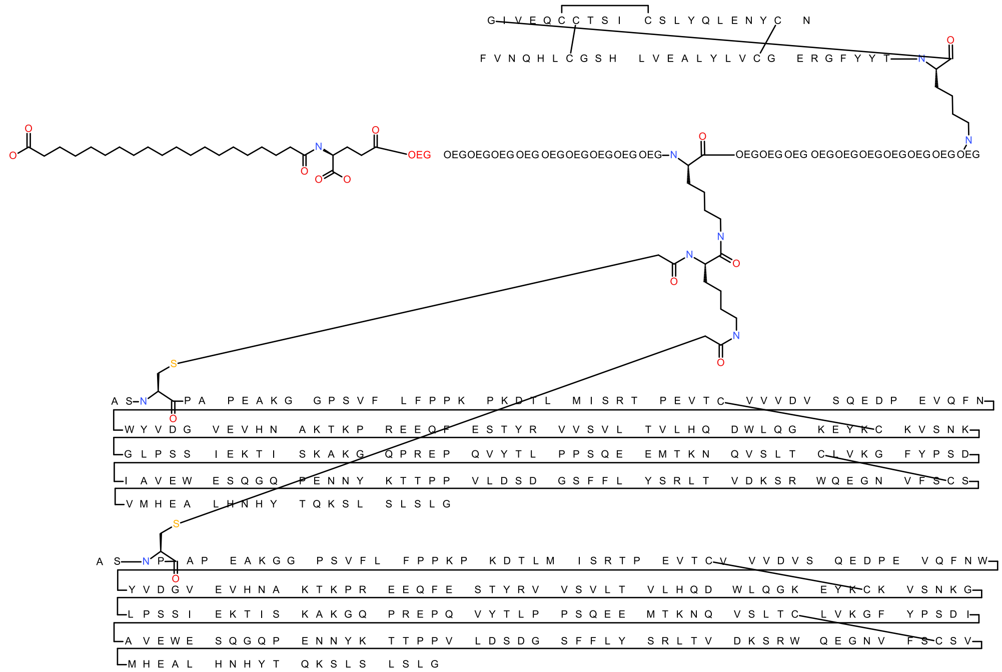

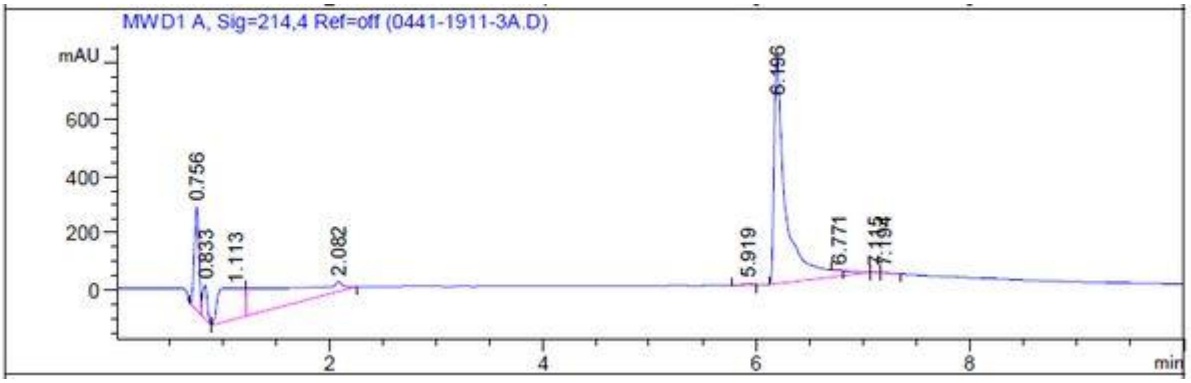


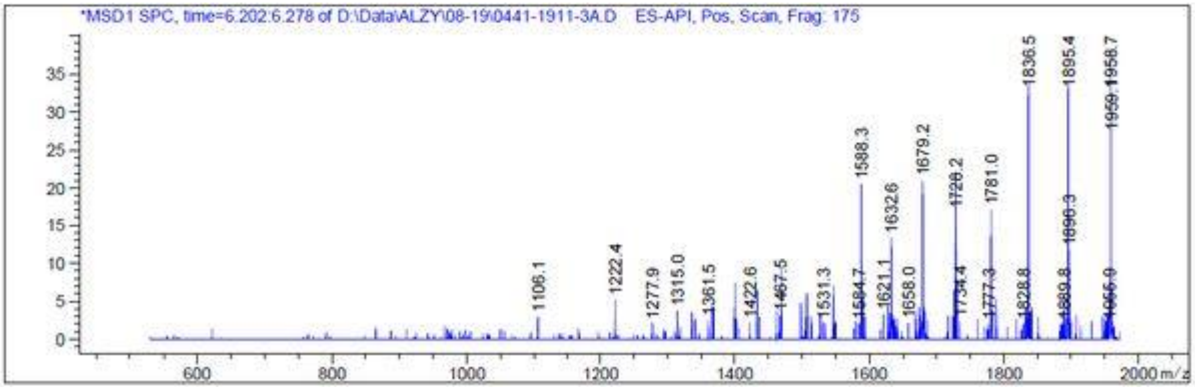


MW exp 58736, obs 58740

Fc-Ins9

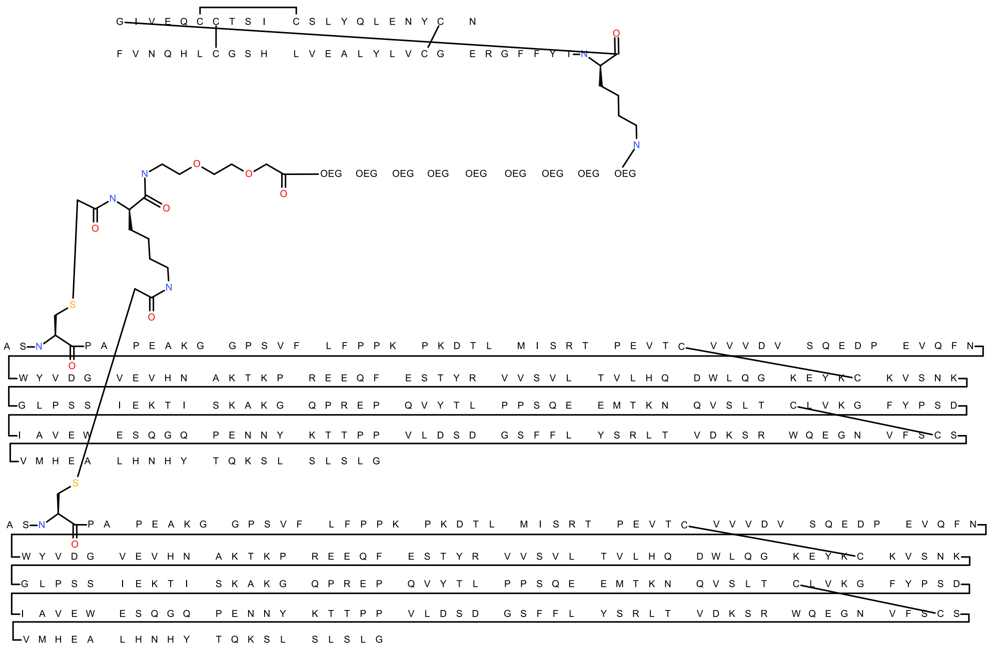

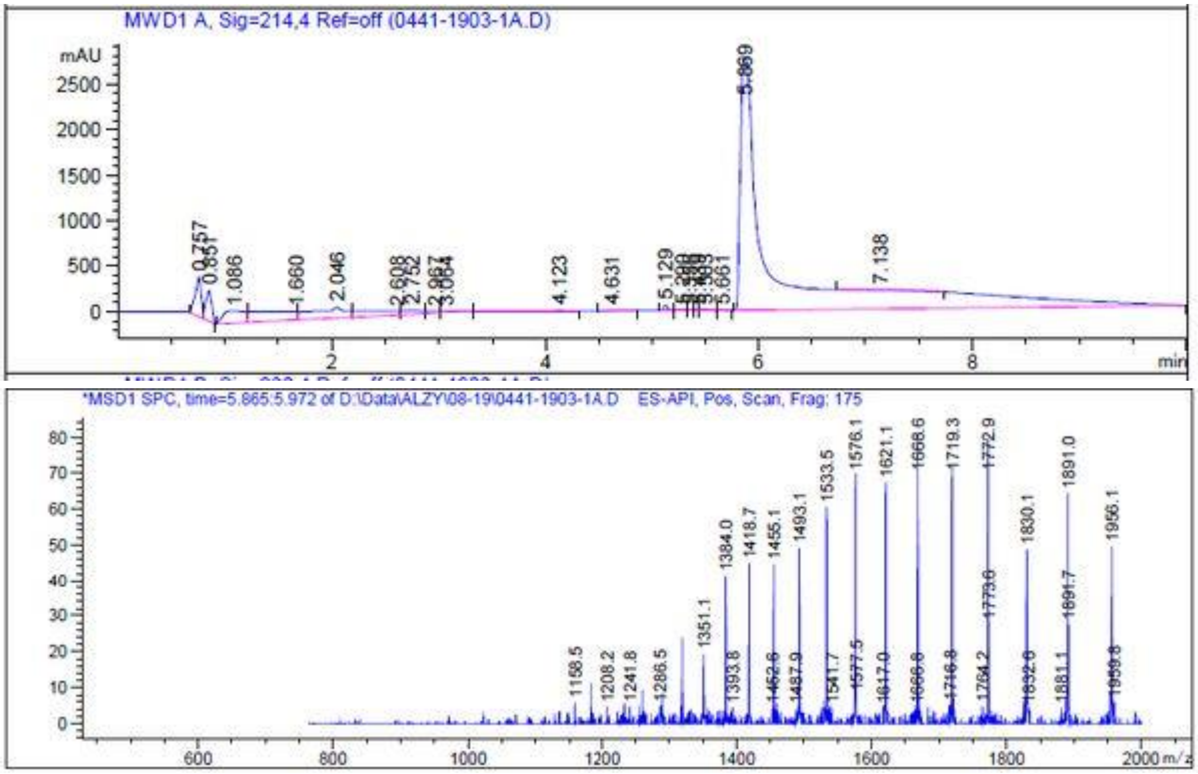


MW exp 56701, obs 56700
